# Effects of pricking-and-cupping bloodletting therapy on pain and skin lesions in patients with herpes zoster: a systematic review and meta-analysis

**DOI:** 10.3389/fneur.2026.1806109

**Published:** 2026-08-26

**Authors:** Pei Peng, Chengting Jiang, Min Wu, Pengcheng Zhong

**Affiliations:** 1 Department of Integrative Medicine, The Central Hospital of Xiangtan (The Affiliated Hospital of Hunan University), Xiangtan, Hunan, China; 2 School of Basic Medical Science, Yunnan University of Chinese Medicine, Kunming, China; 3 Pharmacy Department, Traditional Chinese Medicine Hospital of Bobai County, Bobai, Guangxi, China

**Keywords:** bloodletting, herpes zoster, meta-analysis, pricking bloodletting, pricking-and-cupping bloodletting, randomized controlled trial, snake-like sores

## Abstract

**Objective:**

To systematically assess the effectiveness and safety of pricking-and-cupping bloodletting therapy in relieving pain and improving skin lesion outcomes in herpes zoster (HZ) patients.

**Methods:**

All randomized controlled trials (RCTs) exploring the therapy’s impact on HZ-related pain and skin lesions were identified through a comprehensive electronic search of CNKI, VIP, Wanfang, SinoMed, PubMed, EMBASE, Web of Science, and the Cochrane Library, with the search period ranging from each database’s establishment until November 2025. Included studies were assessed for quality via Cochrane Risk of Bias tool, followed by meta-analysis with Stata MP 19.5. The evidence certainty for each outcome was graded via the GRADE framework. PROSPERO Registration: CRD420251267768。.

**Results:**

A total of 31 studies involving 2,443 patients were ultimately included in the meta-analysis. The pooled analysis demonstrated that, compared with conventional Western medicine, pricking-and-cupping bloodletting therapy yielded superior outcomes across multiple endpoints. These included Visual Analogue Scale (VAS) pain scores [SMD = −1.59, 95% CI (−2.13, −1.05), *p* < 0.001], overall response rate [RR = 1.12, 95% CI (1.04, 1.19), *p* = 0.001], time to cessation of vesicles [SMD = −0.96, 95% CI (−1.34, −0.58), *p* < 0.001], time to crust formation [SMD = −1.29, 95% CI (−1.74, −0.84), *p* < 0.001], time to pain relief [SMD = −5.93, 95% CI (−6.62, −5.25), *p* < 0.001], and the incidence of postherpetic neuralgia (PHN) [RR = 0.14, 95% CI (0.07, 0.27), *p* < 0.001].

**Conclusion:**

Pricking-and-cupping bloodletting therapy shows a relative benefit in shortening the time to pain relief, accelerating skin lesion healing, and reducing the incidence of postherpetic neuralgia (PHN) in patients with herpes zoster. However, given the very low certainty of evidence as assessed by the GRADE framework, clinical interpretation should be cautious. Rigorous, large-sample, multicenter randomized controlled trials are urgently needed to validate its efficacy and strengthen the reliability of clinical evidence.

**Systematic review registration:**

https://www.crd.york.ac.uk/PROSPERO/search, identifier CRD420251267768.

## 1 Introduction

Herpes zoster (HZ), resulting from the reactivation of the varicella-zoster virus (VZV), is a dermatological disorder. It clinically manifests as clustered vesicular eruptions distributed along unilateral dermatomes in a belt-like pattern accompanied by neuropathic pain, typically not crossing the midline of the body (1). In traditional Chinese medicine (TCM), it corresponds to the categories of “she chuan chuang” and “chan yao huo dan” (2). Epidemiological data indicate an incidence rate of 6.64 per 1,000 person-years. The average hospitalization cost per episode for patients with first-onset HZ is approximately RMB 8,116.9, while the average outpatient cost is RMB 560.2. Patients with recurrent HZ have more outpatient visits than those with first-onset disease, and their length of hospital stay is approximately 2.4 days longer (3). HZ predominantly affects individuals aged over 50 years and immunocompromised populations. It can induce a variety of complications, among which postherpetic neuralgia (PHN) is the most intractable. PHN is clinically defined as persistent neuropathic pain (characterized by burning, electric shock-like, or stabbing sensations, as well as allodynia) in the affected dermatome lasting for ≥3 months after the complete resolution and crusting of herpetic skin lesions (4). Additionally, patients may suffer from neurological impairments, including meningitis, bladder dysfunction, and retinal necrosis (4).

Neuropathic pain associated with HZ is classified as a complex neuropathic pain syndrome, which is jointly influenced by disease staging, multiple pain components, and sex-differential immunoregulatory mechanisms. Persistent pain severely impairs patients’ sleep, mood, and activities of daily living. Monotherapy or minimally invasive interventions often yield suboptimal long-term analgesic efficacy. Accumulating clinical and mechanistic evidence supports the use of multimodal combined interventions for refractory HZ neuropathic pain. Non-pharmacological therapies utilizing peripheral physical modulation, such as transcutaneous electrical nerve stimulation (TENS), have been shown to alleviate chronic pain by targeting neuroinflammatory pathways. This suggests that local targeted physical stimulation represents a viable strategy for optimizing pain management in HZ (5–7), thereby providing a theoretical rationale for conducting evidence-based evaluations of local external therapies, such as pricking-and-cupping bloodletting, in this study.

However, current clinical management of HZ predominantly relies on conventional Western medicine and invasive interventional procedures, both of which possess inherent limitations. Pharmacological interventions—including antiviral agents (acyclovir, famciclovir, valacyclovir), nonsteroidal anti-inflammatory drugs (NSAIDs), anticonvulsants, and opioids—are frequently associated with severe adverse effects, such as acute kidney injury, laryngeal paralysis, and systemic tremors (8–10). Similarly, interventional analgesic techniques present significant drawbacks. Nerve ablation can lead to localized numbness, hypoesthesia, and irreversible nerve damage (11, 12). Nerve blocks carry risks of puncture-related hematomas, pneumothorax, and localized infections (13). Central neuromodulation is technically demanding, entails high costs, and may result in complications like cerebrospinal fluid leakage and electrode displacement (14). Furthermore, peripheral minimally invasive neuromodulation often yields suboptimal long-term analgesia and a high incidence of transient local burning or soreness postoperatively, rendering it intolerable for some patients requiring repeated treatments (5, 15). Consequently, there is an urgent clinical need for safe, convenient, and cost-effective complementary alternative therapies.

Pricking-and-cupping bloodletting therapy aligns perfectly with these clinical demands. As a classic local external therapy in traditional Chinese medicine (TCM), it is characterized by simplicity, affordability, and efficacy. It has been widely applied in the management of various pain syndromes (16–18) and is frequently utilized as a clinical treatment for HZ (19). This therapy involves pricking the skin with a three-edged needle or plum-blossom needle to induce localized bleeding, followed by cupping to extract a specific volume of blood (20). According to TCM theory, this modality adheres to the core principle of “removing blood stasis” from the *Yellow Emperor’s Inner Canon*, thereby unblocking local stagnation, regulating disordered visceral Qi, and restoring Yin-Yang homeostasis (21). Existing clinical studies (22–24) suggest that pricking-and-cupping bloodletting may alleviate varicella-zoster virus (VZV)-associated pain by modulating three primary pathways: neuroinflammation, cytokine secretion, and peripheral nerve repair. However, there remains a paucity of preclinical studies directly validating its effects on glial activation and myelin preservation. Thus, the underlying molecular mechanisms require further elucidation, and current evidence provides only preliminary modern pathological support for its clinical application. Upon reactivation, VZV induces a synergistic neuroimmune injury involving both the peripheral and central nervous systems. The massive release of pro-inflammatory cytokines, coupled with peripheral nerve demyelination, drives persistent pain sensitization, which ultimately culminates in PHN (25).

Existing studies have preliminarily confirmed that pricking-and-cupping bloodletting therapy possesses a certain degree of efficacy and safety in the treatment of HZ (26, 27). However, previous systematic reviews on this topic were published relatively early, and a substantial number of new randomized controlled trials have emerged in this field in recent years. Moreover, prior evidence-based studies generally suffered from limitations such as small sample sizes, single outcome assessments, and a lack of timely evidence updates, which are insufficient to meet the current needs of clinical decision-making. Therefore, this study was conducted in accordance with the PRISMA guidelines (28) to perform a systematic review and meta-analysis, comprehensively incorporating relevant randomized controlled trials. The Visual Analog Scale (VAS) pain score was used as the primary outcome to evaluate changes in pain intensity before and after treatment. Secondary outcomes included the overall clinical response rate, blister resolution time, crusting time, pain relief time, incidence of postherpetic neuralgia, and adverse events. Given the heterogeneity among included studies in terms of baseline pain severity, VAS measurement methods, and follow-up duration, this study did not adopt a single fixed VAS reduction threshold as a criterion for treatment response. Instead, pain relief was assessed primarily by comparing the changes in VAS scores between the pricking-and-cupping group and the control group. This study aims to update previous evidence-based conclusions, address gaps in the current evidence base, and objectively identify the methodological shortcomings and evidence limitations of existing original studies, thereby providing evidence-based references for the standardized clinical application of pricking-and-cupping bloodletting therapy.

## 2 Materials and methods

This systematic review adhered to the PRISMA guidelines and checklist. An *a priori* protocol was prospectively registered in the International Prospective Register of Systematic Reviews (PROSPERO) (No.: CRD420251267768).

### 2.1 Inclusion criteria

(1) Participants: patients diagnosed with HZ; (2) No restrictions on nationality, sex, or age; (3) Interventions: Experimental group received pricking-and-cupping bloodletting ± Western medicine, while control group received Western medicine alone; (4) Outcomes: At least one of the following variables was reported: Primary outcome: VAS pain score (a 10 cm ruler, with 0 at the left end indicating no pain and 10 at the right end indicating the most severe pain imaginable; patients mark the corresponding position on the ruler based on the degree of spontaneous pain/tenderness in the affected skin area over the past 24 h, and the measured distance represents the pain score). Secondary outcomes: Overall response rate ([number of cured cases + number of markedly effective cases + number of effective cases] / total number of cases × 100%); Time to cessation of vesicles: Defined as the number of natural days from the initial appearance of erythematous papules to the cessation of new eruptions (i.e., no new papules, vesicles, or papulovesicles); Time to crust formation: Defined as the total number of days from disease onset until all skin lesions (original vesicles and erythematous papules) became completely dry and formed intact yellowish-brown hard crusts, with no residual exudate, erosion, or fresh vesicles; Time to pain relief: Defined as the total number of days from disease onset until the complete resolution of spontaneous pain, allodynia, burning, and stabbing sensations in the affected area, with a sustained VAS score of 0 for at least 7 consecutive days; Incidence of PHN: Defined as the proportion of patients experiencing persistent neuropathic pain (characterized by burning, electric shock-like, or stabbing sensations, as well as allodynia) in the affected dermatome for ≥3 months after the complete resolution and crusting of herpetic skin lesions, expressed as a percentage of the total study population. Safety Outcome: Adverse events (AEs): All intervention- or drug-related AEs, as well as any newly occurring adverse events, were recorded throughout the study. The type, number of cases, incidence rate, severity, and outcomes of these AEs were systematically documented and analyzed.

### 2.2 Exclusion criteria

(1) Duplicate publications; (2) Absence of specific outcome evaluation indices; (3) Reviews, animal experiments, or experience-based descriptions; (4) Full text unavailable; (5) Unclear treatment duration in either the intervention or control group, or inconsistent treatment duration between groups; (6) Errors in data reporting or methodological design; (7) Conference abstracts or clinical practice guidelines; (8) Publications not written in Chinese or English.

### 2.3 Literature search

A comprehensive computerized search was performed in the following Chinese and English databases: CNKI, VIP, Wanfang Data Knowledge Service Platform, SinoMed, PubMed, EMBase, Web of Science, and the Cochrane Library. The search spanned the period from earliest available records in each database until November 2025. Chinese search terms included: “pricking-and-cupping bloodletting,” “cupping with bloodletting,” “pricking,” “bloodletting,” “herpes zoster,” “zona,” “zoster,” “shingles,” “spider nevus-like lesions,” “snake-like herpes,” “fire toxin erythema,” “serpiginous herpes,” “lumbar herpes zoster,” “fire belt herpes,” “snake erythema,” “nest-like herpes,” and “steaming-belt herpes.” English search terms included: “bloodletting,” “pricking blood treatment,” “pricking-cupping,” “cupping,” “herpes zoster,” “zoster,” and “randomized controlled trials.” The detailed search strategy is provided in Supplementary Table 1.

### 2.4 Study selection and data extraction

Two investigators (PP and WM) independently screened the literature and extracted relevant data. After the importation of all retrieved records into EndNote (version 2025–2) and removal of duplicates, the screening procedure began with an initial review of titles/abstracts to rule out irrelevant studies. This was followed by a full-text evaluation of articles that appeared potentially eligible to determine which ones would be finally included. Data regarding critical study characteristics (e.g., first author, publication year, country or region, sample size, age, sex, interventions, controls, and outcome variables) were collected with a standardized form. Cross-checking ensured data accuracy. Resolution of any disagreements was achieved by consulting a third senior investigator.

### 2.5 Quality assessment

For all studies in the analysis, their methodological quality was examined by the Cochrane Risk of Bias Tool as per the Cochrane Handbook 5.1.0. This assessment involved evaluating seven key domains: random sequence generation, allocation concealment, blinding of participants and personnel, blinding of outcome assessment, incomplete outcome data, selective reporting, and other bias (29).

### 2.6 Statistical analysis

The meta-analysis via Stata MP 19.5 involved the calculation of pooled effect estimates. For continuous and dichotomous outcomes, the standardized mean difference (SMD) and relative risk (RR) were computed, respectively, both reported with 95% confidence intervals (CIs). The *I*^2^ statistic served to evaluate heterogeneity, with model selection (fixed-effects for *I*^2^ < 50%; random-effects for *I*^2^ ≥ 50%) dependent on its value. To explore heterogeneity, subgroup analyses were stratified by intervention type and treatment duration, alongside meta-regression analyses incorporating treatment course, publication year, and sample size. A leave-one-out sensitivity analysis was implemented to verify the stability of the findings. Publication bias assessment followed a tiered approach. For meta-analyses with ≥10 included studies, a funnel plot combined with Egger’s regression test was used (non-significant bias if *p* > 0.05); for <10 studies, Egger’s test was underpowered, thus only a funnel plot was generated for visual trend assessment without quantitative testing. If the funnel plot indicated clear asymmetry, only the trim-and-fill method was used for sensitivity correction, and the results were interpreted with extreme caution.

### 2.7 Assessment of certainty of evidence using GRADE

Evidence certainty for each outcome was rated with GRADE Profiler 3.6. Since all studies were RCTs, the evaluation considered five domains: publication bias, inconsistency, indirectness, imprecision, and study limitations. The certainty of evidence was rated as high, moderate, low, or very low.

## 3 Results

### 3.1 Literature screening process and results

**Figure 1:**
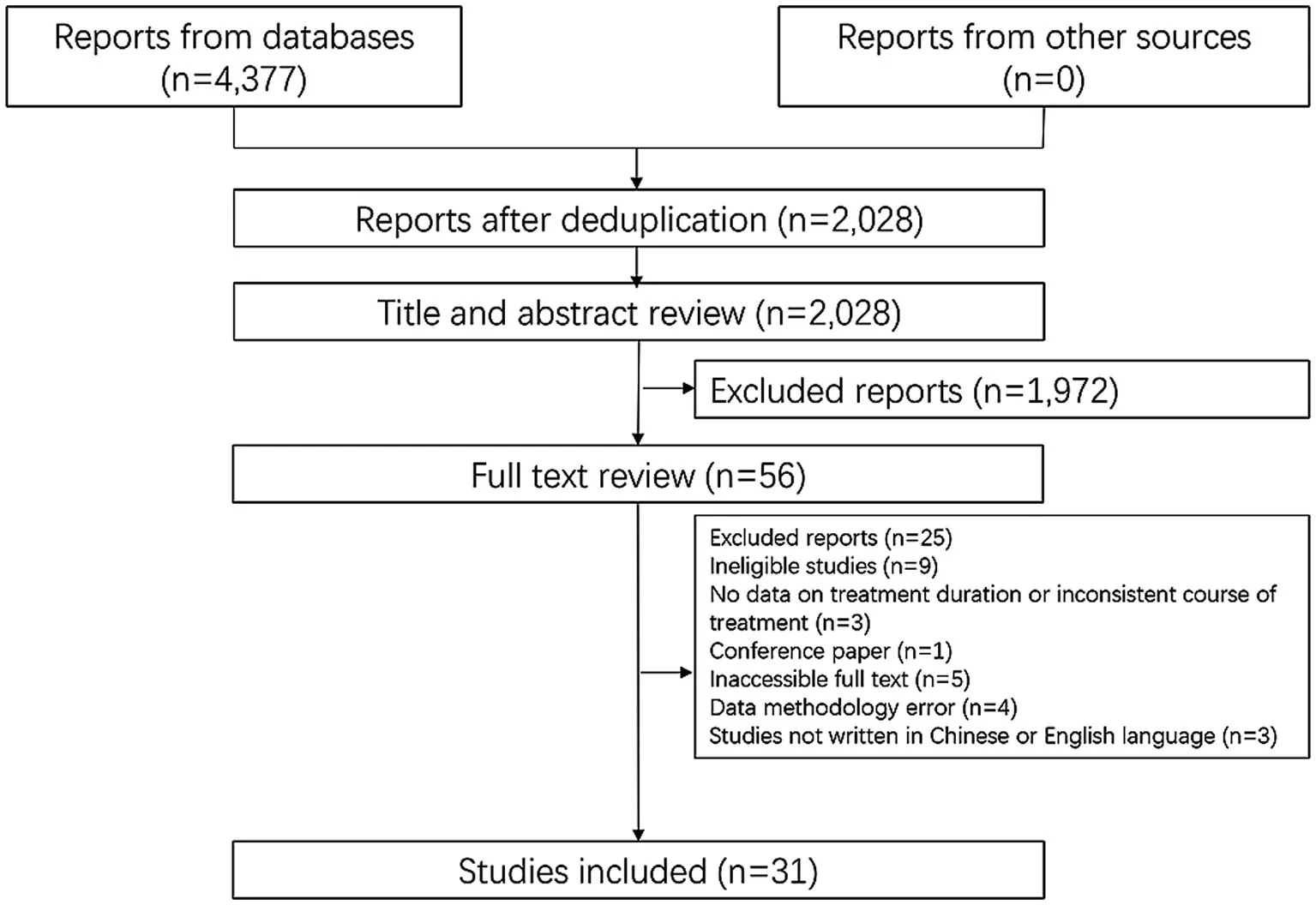
Flowchart of literature screening for studies on pricking-and-cupping bloodletting therapy for the treatment of herpes zoster.

A total of 4,377 records were found via the predefined search and data collection (CNKI: 1,156; Wanfang: 1,299; VIP: 742; SinoMed: 961; PubMed: 49; Cochrane: 51; EMBASE: 68; Web of Science: 51). After deduplication of 2,349 duplicate entries, title/abstract screening excluded 381 non-RCTs (reviews, experience-based studies, conference papers, and guidelines), 2 animal studies, and 1,589 topic-irrelevant records. Full-text screening of 56 remaining articles excluded nine ineligible, three with unclear treatment duration, one conference paper, five unavailable texts, four with data/method errors, and three non-Chinese/English studies. Final inclusion: 31 (30–60) Chinese-language RCTs. The PRISMA flow diagram is set out in Figure 1.

### 3.2 Characteristics of included studies

Among the 31 RCTs, one study (36) contributed data from two intervention arms, totaling 2,443 participants (1,236 in experimental groups; 1,207 in controls). Regarding intervention details, Combined therapy (pricking-and-cupping bloodletting + Western medicine): 18 studies (30, 31, 33–39, 41, 42, 44, 47–50, 58, 60) monotherapy (pricking-and-cupping bloodletting alone): 14 studies (32, 36, 40, 43, 45, 46, 51–57, 59). The volume of blood extracted during pricking-and-cupping bloodletting ranged from 2 to 20 mL, with most studies reporting an extraction volume of 3 to 10 mL. Some studies defined the endpoint simply as the presence of oozing blood or the drainage of vesicular fluid. The retention time for a single cupping session spanned from 1 to 15 min, typically 3 to 10 min. Treatment frequency was generally prescribed as once daily or once every other day, with treatment courses lasting from 7 to 30 days, predominantly 7 to 14 days. The control group interventions consisted of conventional Western medicine, including oral or intravenous antiviral agents, neurotrophic agents (e.g., mecobalamin), analgesics (e.g., gabapentin and pregabalin), glucocorticoids, B-complex vitamins, topical antiviral ointments, and interferon injections. Dosages, administration frequencies, and routes of administration varied across the study protocols. Further details are provided in Supplementary Table 2. Regarding outcome reporting; VAS scores: 11 studies (30, 32, 34, 36–39, 41, 42, 46, 51); overall response rate: 27 studies (30, 31, 33–35, 37, 38, 40, 41, 43–60) (The specific diagnostic criteria used to determine the overall response rate in each study are detailed in Supplementary Table 3); time to cessation of vesicles: six studies (37, 47, 49, 50, 54, 58); time to crust formation: seven studies (32, 38, 41, 47, 49, 50, 58); time to pain relief: two studies (47, 49); incidence of PHN: eight studies (30, 34, 37, 41, 46, 57–59); and adverse reactions: six studies (30, 31, 36, 41, 45, 53). Study characteristics are set forth in Table 1.

**Table 1:** Characteristics of studies included in the meta-analysis of pricking-and-cupping bloodletting therapy for HZ.

Included studies	Year	Sample size	Intervention		Treatment duration	Outcomes
		Experiment/control	Experiment	Control		
Shu Liu (30)	2025	30/30	Pricking-and-cupping bloodletting therapy + Western medicine	Western medicine	14d	①②⑥⑦
Liang Li (31)	2025	50/50	Pricking-and-cupping bloodletting therapy + Western medicine	Western medicine	12d	①⑦
Xingxing Gai (32)	2024	50/50	Pricking-and-cupping bloodletting therapy	Western medicine	14d	②④
Mi Wang (33)	2023	30/30	Pricking-and-cupping bloodletting therapy + Western medicine	Western medicine	7d	①
Hui Li (34)	2022	32/32	Pricking-and-cupping bloodletting therapy + Western medicine	Western medicine	30d	①②⑥
Lei Cao (35)	2022	30/30	Pricking-and-cupping bloodletting therapy + Western medicine	Western medicine	7d	①
Xiaogang Zhang (36)	2021	30/31	Pricking-and-cupping bloodletting therapy + Western medicine	Western medicine	7d	②⑦
Xiaogang Zhang (36)	2021	32/31	Pricking-and-cupping bloodletting therapy	Western medicine	7d	②⑦
Jiajun Shi (37)	2021	34/34	Pricking-and-cupping bloodletting therapy + Western medicine	Western medicine	14d	①②③⑥
Zhiyang Meng (38)	2021	36/35	Pricking-and-cupping bloodletting therapy + Western medicine	Western medicine	7d	①②④
Yufeng Wu (39)	2020	30/30	Pricking-and-cupping bloodletting therapy + Western medicine	Western medicine	15d	②
Gang Wang (40)	2020	54/48	Pricking-and-cupping bloodletting therapy	Western medicine	7d	①
Jing Cui (41)	2020	30/30	Pricking-and-cupping bloodletting therapy + Western medicine	Western medicine	7d	①②④⑥⑦
Guilin Jiang (42)	2019	32/31	Pricking-and-cupping bloodletting therapy + Western medicine	Western medicine	15d	②
Xiaoce Cai (43)	2019	16/16	Pricking-and-cupping bloodletting therapy	Western medicine	20d	①
Jian’en Pang (44)	2018	32/32	Pricking-and-cupping bloodletting therapy + Western medicine	Western medicine	14d	①
Zhiquan Li (45)	2018	59/59	Pricking-and-cupping bloodletting therapy	Western medicine	10d	①⑦
Yukui Xing (46)	2017	28/28	Pricking-and-cupping bloodletting therapy	Western medicine	14d	①②⑥
Meng Bai (47)	2017	49/49	Pricking-and-cupping bloodletting therapy + Western medicine	Western medicine	10d	①③④⑤
Fan Wei (48)	2016	30/34	Pricking-and-cupping bloodletting therapy + Western medicine	Western medicine	21d	①
Laisheng Tong (49)	2016	42/40	Pricking-and-cupping bloodletting therapy + Western medicine	Western medicine	10d	①③④⑤
Hu Li (50)	2014	46/44	Pricking-and-cupping bloodletting therapy + Western medicine	Western medicine	10d	①③④
Wei Chen (51)	2014	30/30	Pricking-and-cupping bloodletting therapy	Western medicine	10d	①②
Jianzhong Gao (52)	2014	30/30	Pricking-and-cupping bloodletting therapy	Western medicine	10d	①
Bohua Zhao (53)	2013	36/36	Pricking-and-cupping bloodletting therapy	Western medicine	14d	①⑦
Dong Pan (54)	2013	32/32	Pricking-and-cupping bloodletting therapy	Western medicine	14d	①③
Haiying Liu (55)	2013	30/30	Pricking-and-cupping bloodletting therapy	Western medicine	7d	①
Suju Shao (56)	2012	28/23	Pricking-and-cupping bloodletting therapy	Western medicine	7d	①
Suyan Song (57)	2011	80/70	Pricking-and-cupping bloodletting therapy	Western medicine	7d	①⑥
Huajun Wu (58)	2009	60/60	Pricking-and-cupping bloodletting therapy + Western medicine	Western medicine	10d	①③④⑥
Yalan Zhang (59)	2006	68/62	Pricking-and-cupping bloodletting therapy	Western medicine	7d	①⑥
Lan Xu (60)	2004	40/40	Pricking-and-cupping bloodletting therapy + Western medicine	Western medicine	7d	①

① overall response rate; ② visual analog scale (VAS) score; ③ time to cessation of vesicles; ④ time to crust formation; ⑤ time to pain relief; ⑥ postherpetic neuralgia; ⑦ adverse reactions.

### 3.3 Methodological quality assessment of included studies

**Figure 2:**
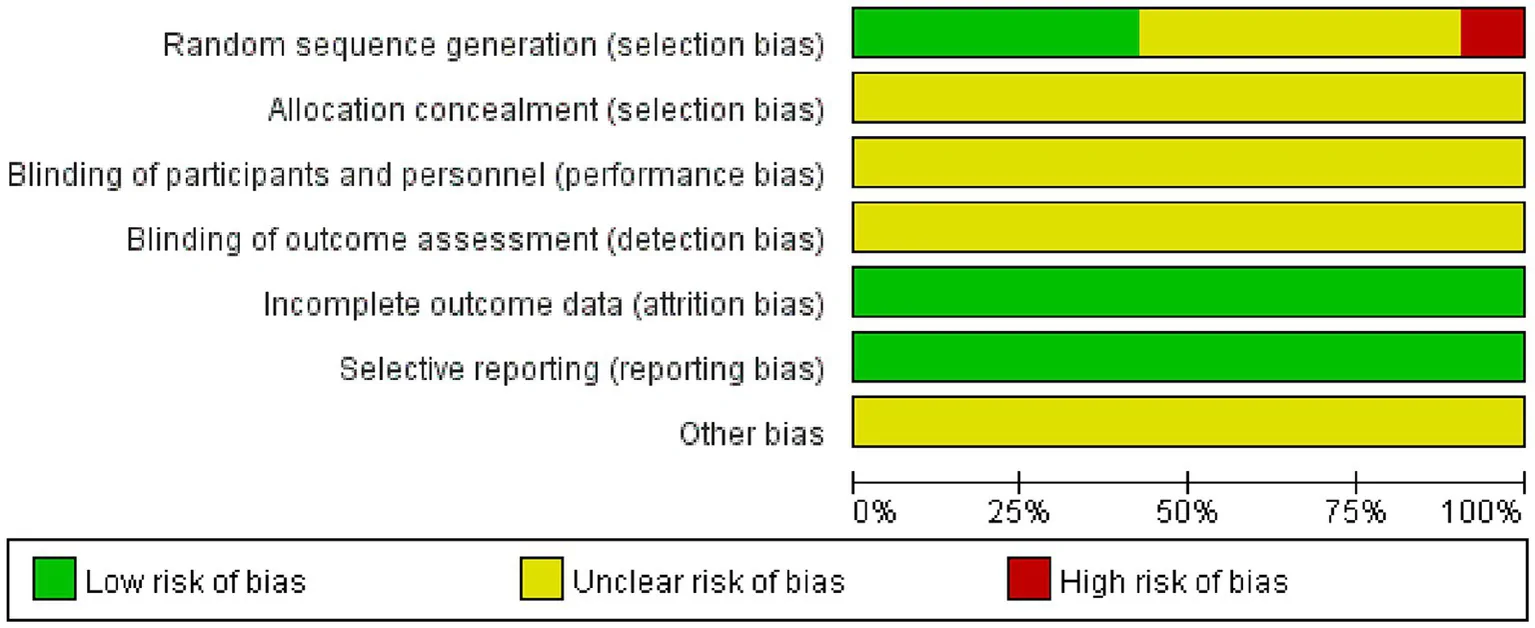
Bar chart of risk of bias distribution of included studies.

**Figure 3:**
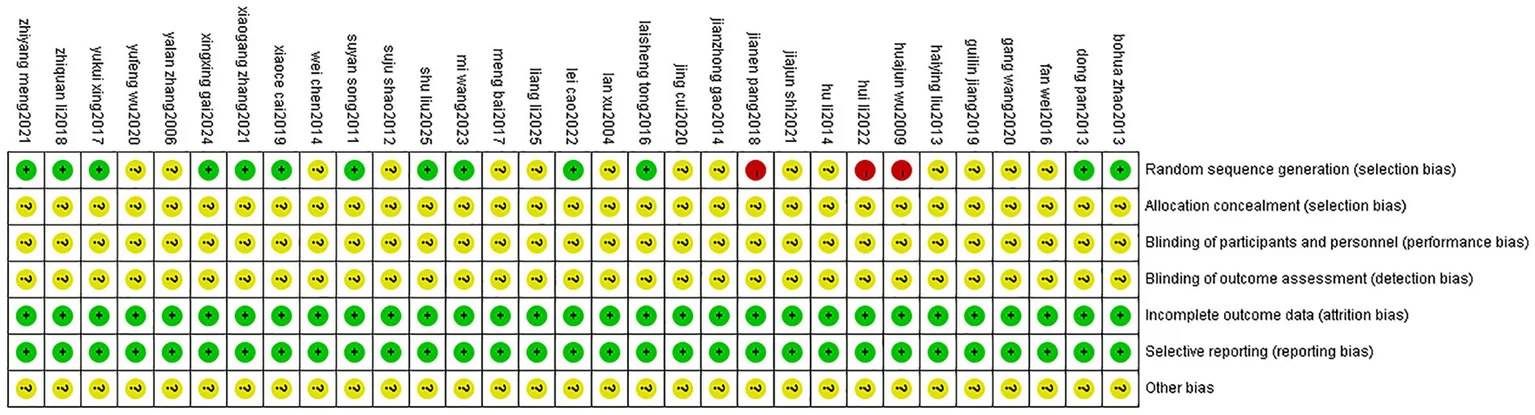
Summary of risk of bias in the included studies.

The Cochrane RoB Tool (5.1.0) was applied to appraise the methodological quality of included studies. (1) Random sequence generation: Ten studies (30, 32, 36, 38, 43, 45, 46, 49, 54, 57) (random number table); one study (33) (random number table); one study (35) (random digit method); one study (53) (lot-drawing method); one study (58) (odd–even day assignment); one study (44) (patient preference); one study (39) (sealed envelopes); one study (34) (0atients were randomly assigned in the order of their clinic visits); and 14 studies (31, 37, 40–42, 47, 48, 50–52, 55, 56, 59, 60) (unspecified randomization method). (2) Allocation concealment: None (30–60) reported. (3) Blinding of participants and personnel: No (30–60) participant/personnel described. (4) Blinding of outcome assessment: No (30–60) assessor blinding. (5) Incomplete outcome data: One study (42) reported exclusions and dropouts (three cases in total) and did not apply intention-to-treat analysis (61), the remaining studies (30–41, 43–60) documented complete outcome data. (6) Selective reporting: None detected. (7) Other bias: Unclear. Quality assessment results are presented in Figures 2, 3.

### 3.4 Meta-analysis results

#### 3.4.1 VAS scores

**Figure 4:**
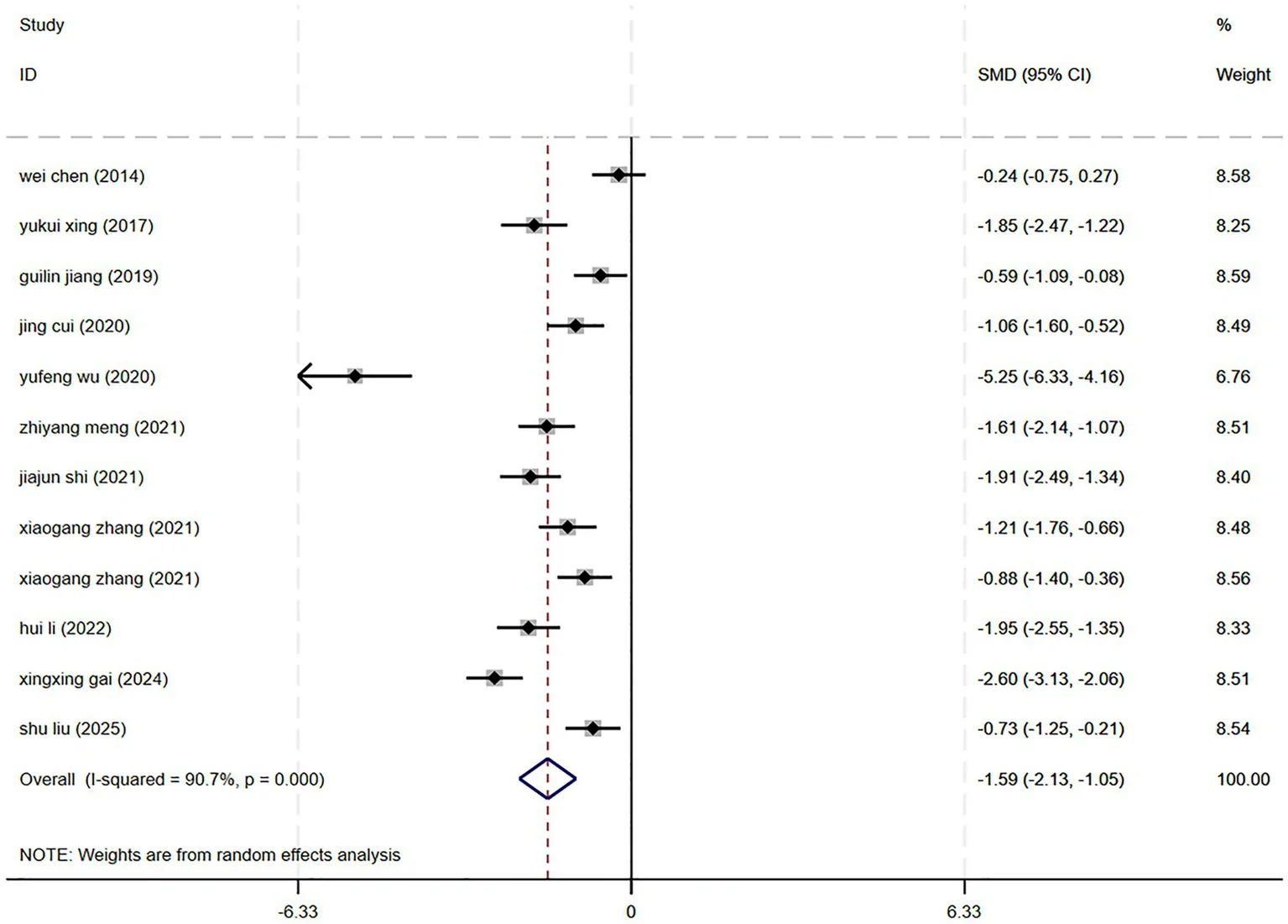
Forest plot of VAS scores (The center square on each horizontal line represents the individual effect size of a single study; the horizontal line extending from both ends of the square indicates the 95% confidence interval (CI) of the effect size). The black diamond at the bottom represents the pooled effect size, with its left and right ends indicating the 95% CI of the pooled effect size. The same legend applies to all subsequent figures.

Data on VAS scores were extracted from 11 studies (30, 32, 34, 36–39, 41, 42, 46, 51), among which one study (36) provided two sets of VAS data, yielding a total of 12 data sets. The heterogeneity test revealed significant heterogeneity across studies (*I*^2^ = 90.7%, *p* < 0.001), and a random-effects model was therefore applied. The results indicated a statistically significant difference in VAS scores between the bloodletting cupping group and the Western medicine group [SMD = -1.59, 95%CI (−2.13, −1.05), *p* < 0.001] (Figure 4).

Substantial inter-study heterogeneity prompted subgroup analyses by intervention type and treatment duration. Patients treated for >7 days had a significantly greater VAS score reduction [SMD = −1.81, 95% CI (−2.75, −0.88), *p* < 0.001], while those with ≤7 days of treatment also showed greater pain relief as reflected by VAS scores in the experimental group versus the control [SMD = −1.33, 95% CI (−1.70, −0.96), p < 0.001]. However, the small number of included studies necessitates cautious interpretation of these findings (Table 2).

**Table 2:** Subgroup analysis of VAS by intervention type and treatment duration.

Subgroup	Category	No. of studies	*n*	SMD	95%CI	I2	Z	P
Intervention	Pricking-and-cupping bloodletting therapy	4	279	−1.39	[−2.44,-0.33]	93.3%	2.58	*p* = 0.01
	Pricking-and-cupping bloodletting therapy + Western medicine	8	507	−1.69	[−2.36,-1.03]	90.5%	4.98	*p* < 0.001
Treatment duration	>7 days	7	467	−1.81	[−2.75,-0.88]	94.5%	3.79	*p* < 0.001
	≤7 days	5	319	−1.33	[−1.70,-0.96]	56.4%	7.02	*p* < 0.001

Meta-regression analyses showed that treatment course (*I*^2^ = 90.75%, *p* = 0.376), publication year (*I*^2^ = 90.46%, *p* = 0.538), and sample size (*I*^2^ = 89.27%, *p* = 0.477) did not significantly moderate the pooled effect size, indicating that none of these variables could explain the substantial heterogeneity observed among studies (see Supplementary Figures 1–3).

Sensitivity analysis demonstrated that the results remained stable after excluding studies with a high risk of bias. Regarding publication bias, Egger’s test yielded a *p*-value of 0.002, indicating significant publication bias. Consequently, the trim-and-fill method was applied for correction. The results showed no substantial reversal of the p-value after correction; however, the Egger test confirmed the presence of significant publication bias, suggesting a potential for selective reporting of positive outcomes and a risk of overestimation of the pooled effect. Clinical interpretation of these findings should therefore be undertaken with caution (see Supplementary Figures 4–8).

#### 3.4.2 Overall response rate

**Figure 5:**
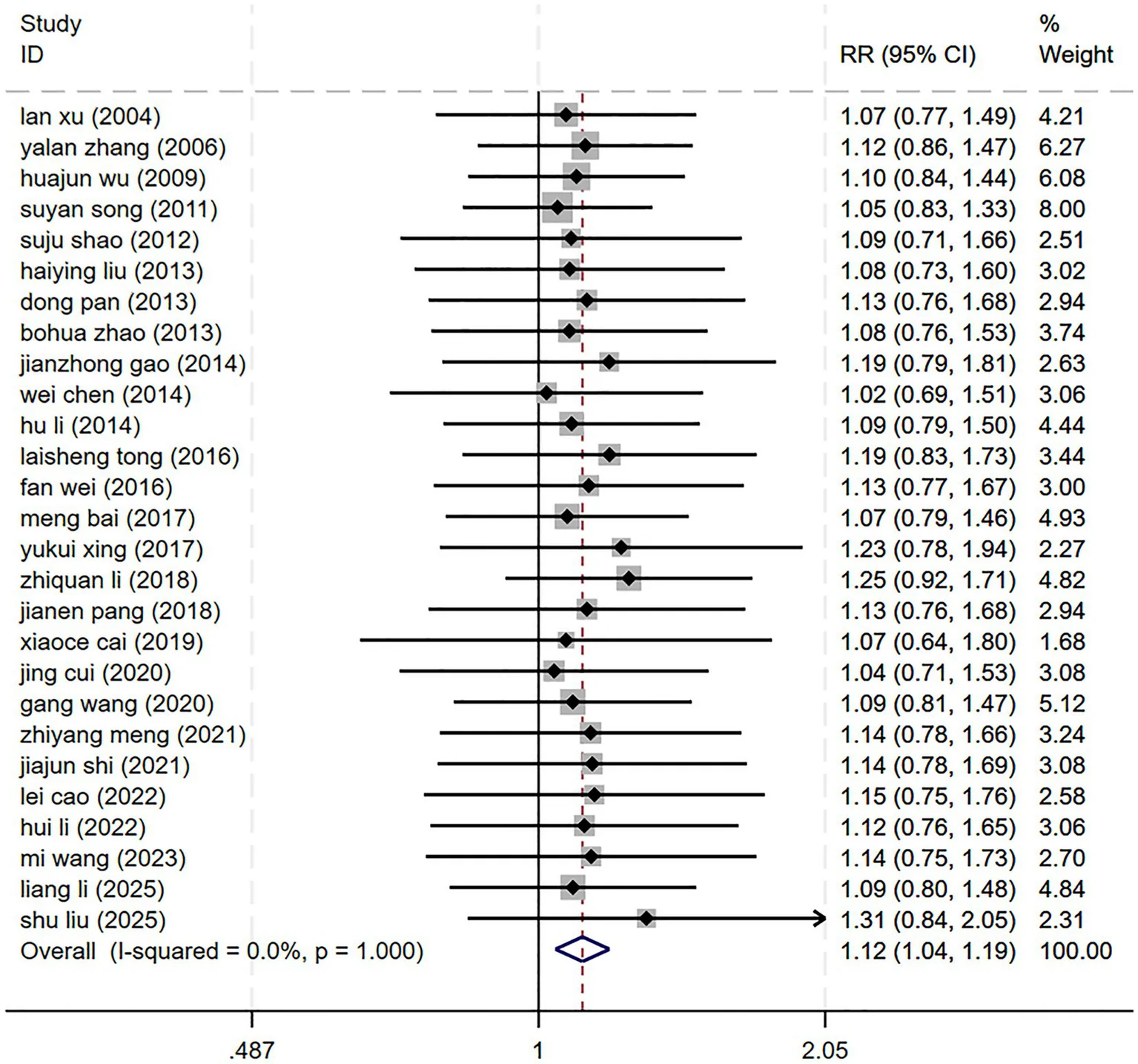
Forest plot of the overall response rate.

A total of 27 studies (30, 31, 33–35, 37, 38, 40, 41, 43–60) reported the overall response rate, involving 2,096 participants. No significant heterogeneity was detected across studies (*I*^2^ = 0.0%, *p* = 1.0), leading to the application of a fixed-effect model. The bloodletting cupping group demonstrated a significantly higher overall therapeutic effect rate than the Western medicine group [RR = 1.12, 95% CI (1.04, 1.19), *p* = 0.001] (Figure 5).

Sensitivity analysis confirmed the stability of these results. Egger’s test for publication bias yielded a *p*-value of 0.081, indicating no significant bias. Further details are provided in Supplementary Figures 9–11.

#### 3.4.3 Time to cessation of vesicles

**Figure 6:**
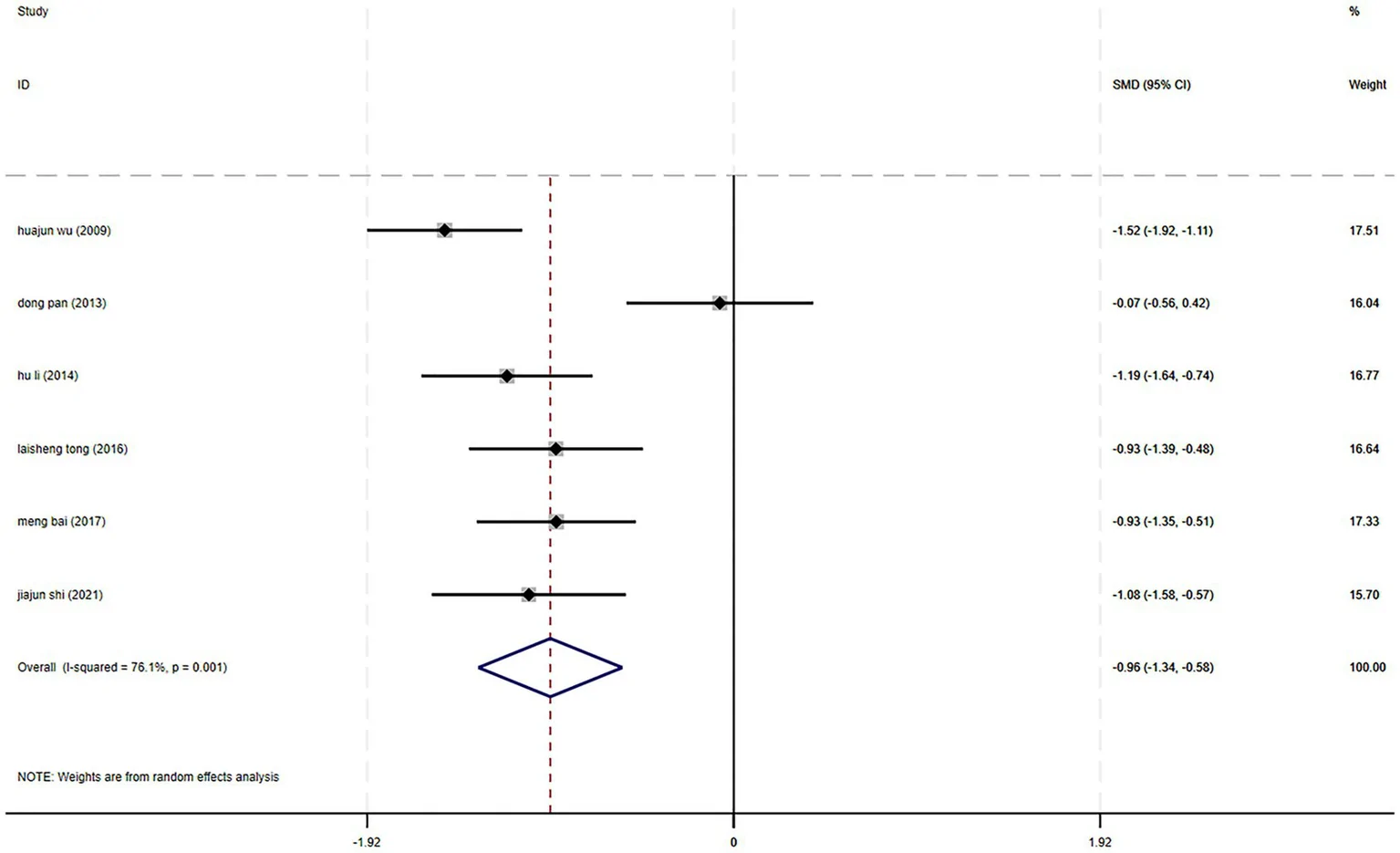
Forest plot of time to cessation of vesicles.

A total of six studies (37, 47, 49, 50, 54, 58) reported time to cessation of vesicles. Heterogeneity testing indicated substantial heterogeneity among studies (*I*^2^ = 71.6%, *p* = 0.001), making a random-effects model necessary. The pooled data demonstrated a statistically significant difference between groups variation (SMD = −0.96, 95% CI − 1.34 to −0.58, *p* < 0.001), favoring the pricking-and-cupping bloodletting therapy group (Figure 6). Given the significant heterogeneity, Subgroup analysis based on interventions and treatment duration showed that in patients with a treatment duration ≤10 days, the time to cessation of vesicles was significantly shorter (SMD = −1.15, 95% CI − 1.43 to −0.87, *p* < 0.001). Detailed results are presented in Table 3.

**Table 3:** Subgroup analysis of time to cessation of vesicles by intervention type and treatment duration.

**Subgroup**	**Category**	**No. of studies**	**n**	**SMD**	**95%CI**	**I2**	**Z**	**P**
Intervention	Pricking-and-cupping bloodletting therapy	1	64	−0.07	[−0.56,–0.42]	/	2.58	*p* = 0.772
	Pricking-and-cupping bloodletting therapy + Western medicine	5	458	−1.14	[−1.37,–0.91]	22.5%	4.98	*p* < 0.001
Treatment duration	>10 days	2	132	−0.57	[−1.55,0.41]	87.1%	1.14	*p* = 0.254
	≤10 days	4	390	−1.15	[−1.43,–0.87]	41.0%	8.02	*p* < 0.001

Meta-regression analysis showed that treatment course (*I*^2^ = 68.77%, *p* = 0.195), publication year (*I*^2^ = 79.66%, *p* = 0.783), and sample size (*I*^2^ = 52.65%, *p* = 0.081) did not significantly moderate the pooled effect size, indicating that none of these variables could explain the substantial heterogeneity observed among studies. Detailed information is provided in Supplementary Figures 12–14.

Sensitivity analysis demonstrated that the results remained stable after excluding studies with a high risk of bias. Funnel plot assessment implied the presence of publication bias, leading to the implementation of the trim-and-fill method. The adjusted results showed no substantial reversal of statistical significance. However, the funnel plot indicated the presence of publication bias, suggesting a potential for selective reporting of positive outcomes and a risk of overestimation of the pooled effect. Clinical interpretation of these findings should therefore be undertaken with caution. Detailed information is provided in Supplementary Figures 15–18.

#### 3.4.4 Time to crust formation

**Figure 7:**
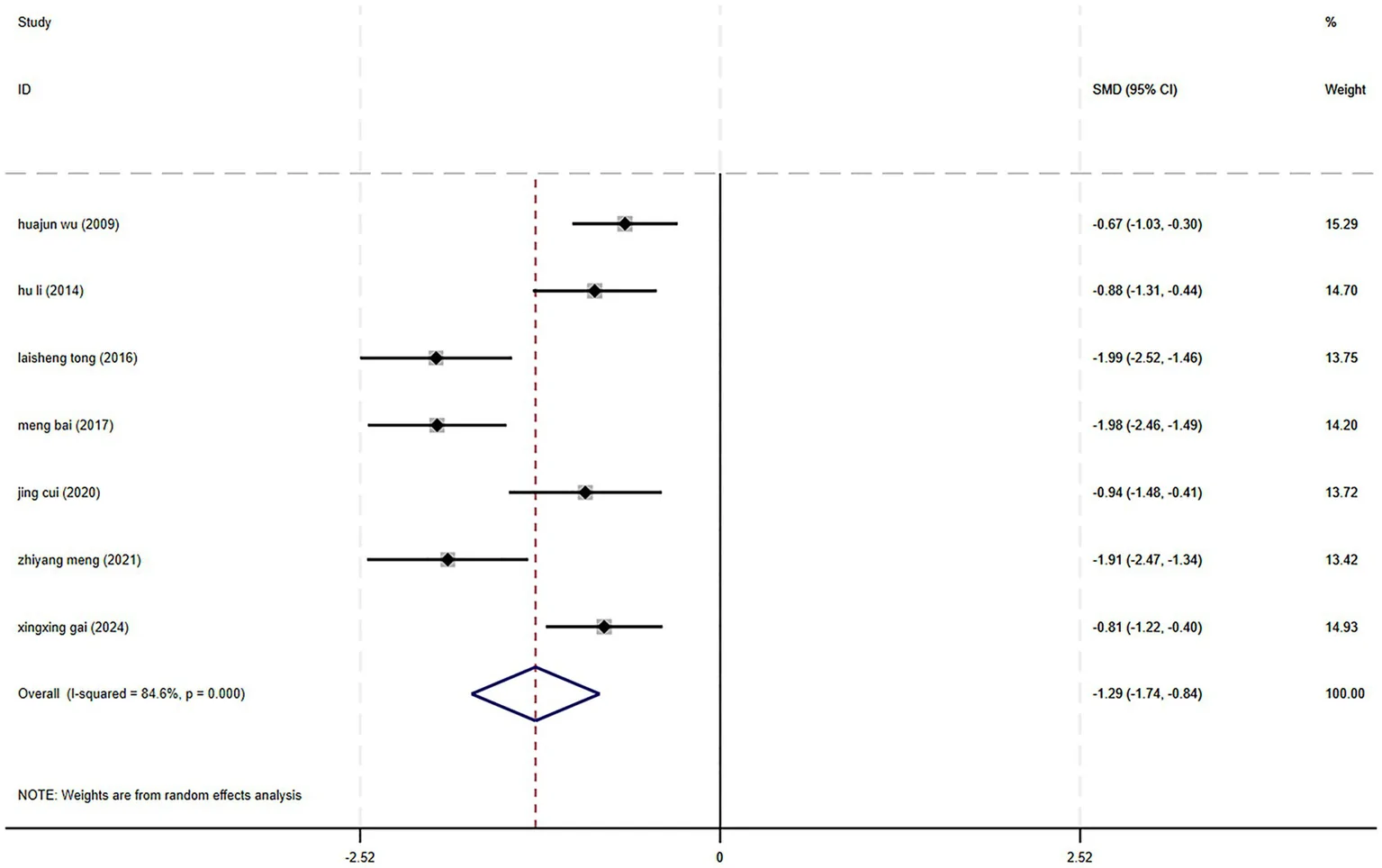
Forest plot of time to crust formation.

Seven studies (32, 38, 41, 47, 49, 50, 58) reported the time to crust formation, including 621 participants (intervention group: 313 and control group: 308). Given the detection of substantial heterogeneity (*I*^2^ = 84.6%, *p* < 0.001), a random-effects model was utilized. The pooled data showed a statistically significant shorter time to crust formation in the pricking-and-cupping bloodletting therapy group (SMD = −1.29, 95% CI − 1.74 to −0.84, *p* < 0.001) (Figure 7).

Given significant inter-study heterogeneity, subgroup analyses based on intervention type and treatment duration indicated that in patients with treatment duration ≤7 days, time to crust formation was significantly shortened (SMD = −1.42, 95% CI − 2.36 to −0.48, *p* = 0.003). In patients with treatment duration >7 days, the intervention group also showed a shorter time to crust formation compared with the control group (SMD = −1.25, 95% CI − 1.80 to −0.69, *p* < 0.001). Nevertheless, given the limited number of studies included in this outcome analysis, these results should be interpreted with caution (Table 4).

**Table 4:** Subgroup analysis of time to crust formation by intervention type and treatment duration.

Subgroup	Category	No. of studies	*n*	SMD	95%CI	I2	*Z*	*P*
Intervention	Pricking-and-cupping bloodletting therapy	1	100	−0.81	[−1.22,-0.40]	/	3.9	*p* < 0.001
	Pricking-and-cupping bloodletting therapy + Western medicine	6	521	−1.38	[−1.89,-0.86]	85.7%	5.24	*p* < 0.001
Treatment duration	>7 days	5	490	−1.25	[−1.8,-0.69]	87.3%	4.43	*p* < 0.001
	≤7 days	2	131	−1.42	[−2.36,-0.48]	83.1%	2.95	*P* = 0.003

Meta-regression analysis showed that treatment course (*I*^2^ = 85.57%, *p* = 0.491), publication year (*I*^2^ = 86.33%, *p* = 0.665), and sample size (*I*^2^ = 84.21%, *p* = 0.453) did not significantly moderate the pooled effect size, indicating that none of these variables could explain the substantial heterogeneity observed among studies. Detailed information is provided in Supplementary Figures 19–21.

Sensitivity analysis demonstrated that the results remained stable after excluding studies with a high risk of bias. Funnel plots suggested potential publication bias; therefore, the trim-and-fill method was applied. No substantial reversal of statistical significance was observed after adjustment, indicating that the results remain reliable. However, the funnel plot indicated the presence of publication bias, suggesting a potential for selective reporting of positive outcomes and a risk of overestimation of the pooled effect. Clinical interpretation of these findings should therefore be undertaken with caution. Detailed information is provided in Supplementary Figures 22–25.

#### 3.4.5 Time to pain relief

**Figure 8:**
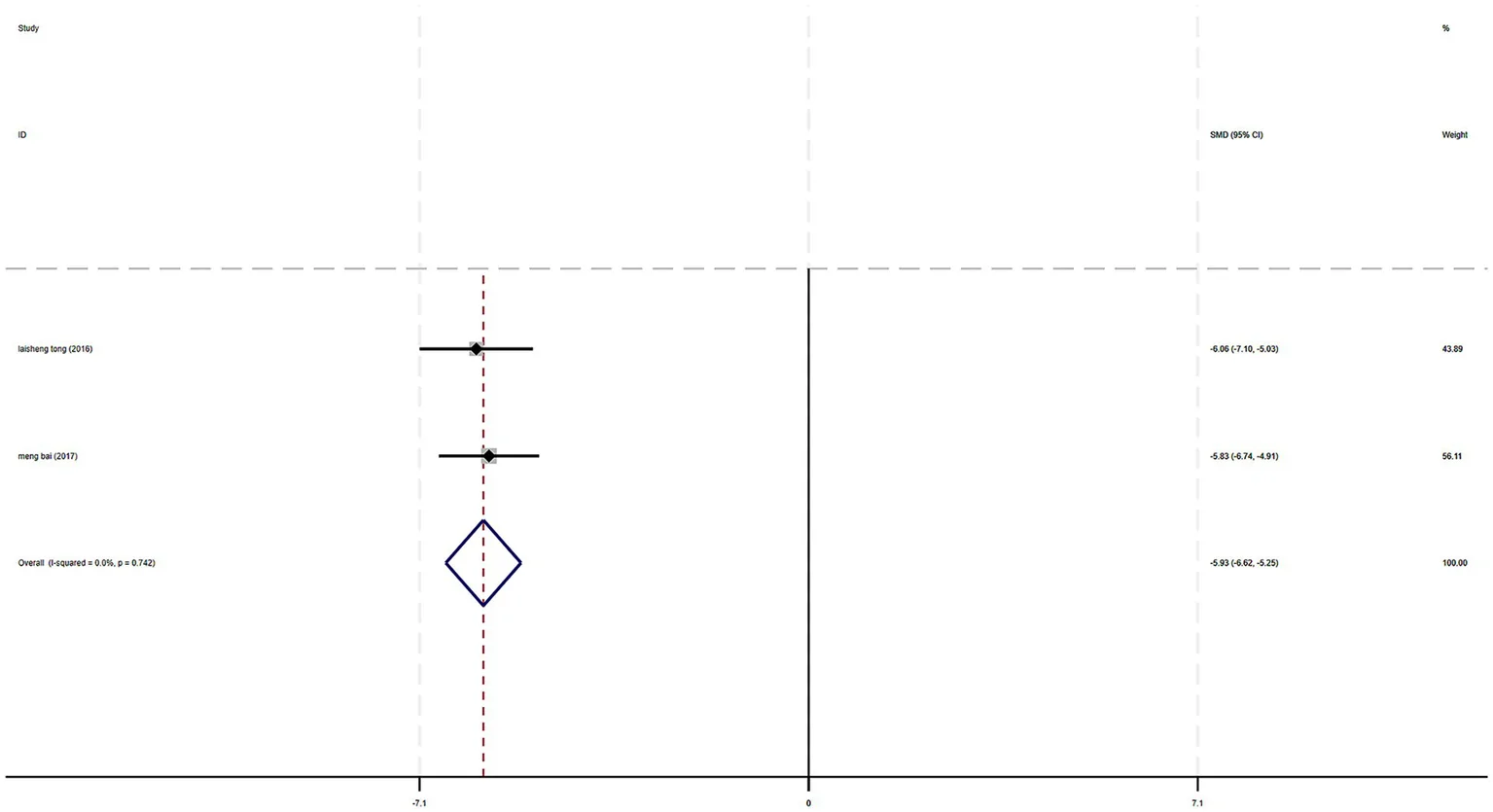
Forest plot of time to pain relief.

Two studies (47, 49) reported time to pain relief, involving 180 participants (intervention group: 91 and control group: 89). Low heterogeneity (*I*^2^ = 0.0%, *p* = 0.742) required the adoption of a fixed-effects model. Pooled data revealed a considerably shorter time to pain relief in the pricking-and-cupping bloodletting therapy group (SMD = −5.93, 95% CI − 6.62 to −5.25, *p* < 0.001; Figure 8).

#### 3.4.6 Incidence of PHN

**Figure 9:**
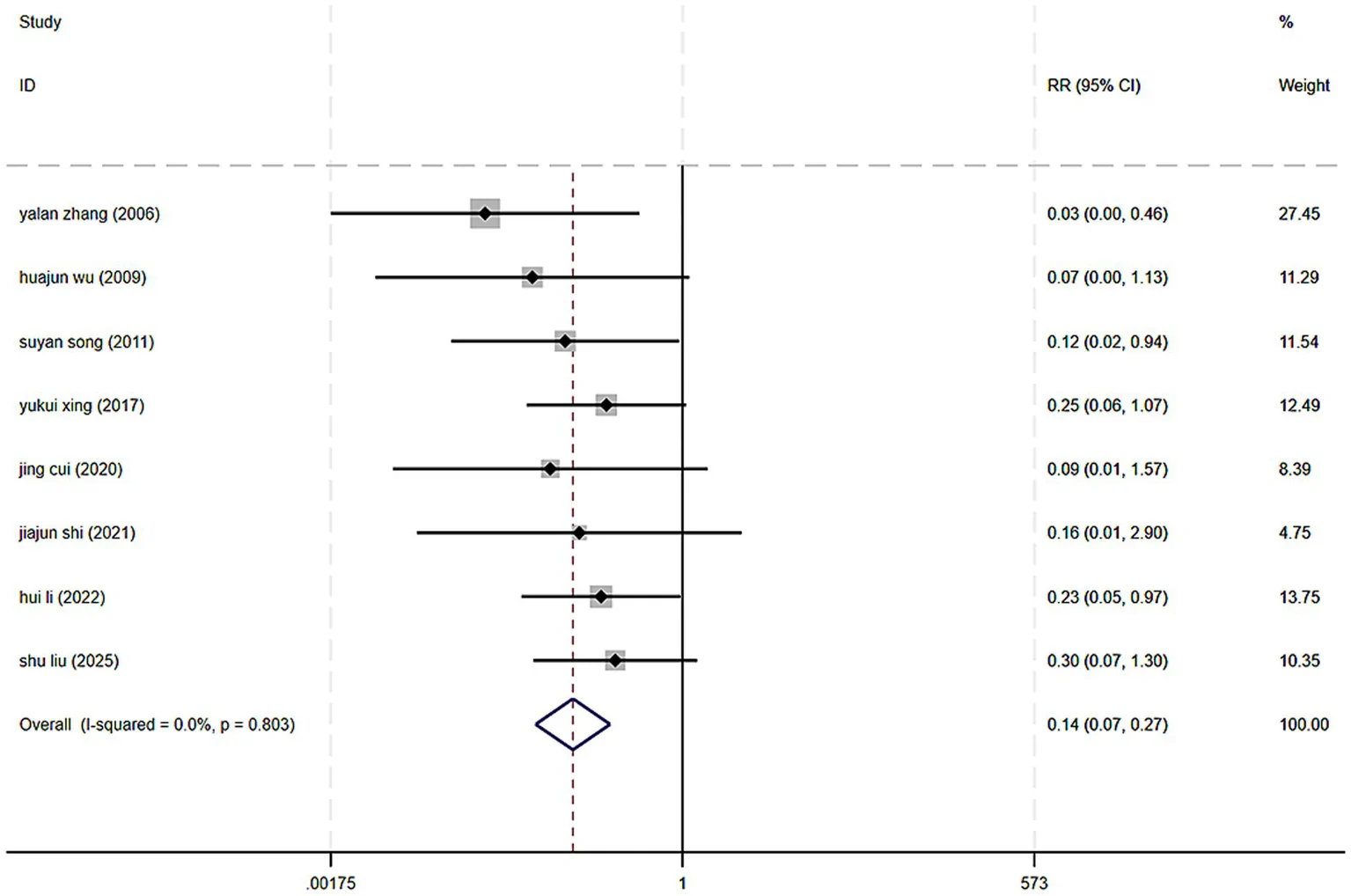
Forest plot of the incidence of PHN.

Eight studies (30, 34, 37, 41, 46, 57–59) reported the incidence of PHN, including 708 participants (intervention group: 362 and control group: 346). Heterogeneity test indicated good homogeneity (*I*^2^ = 0.0%, *p* = 0.803), leading to the application of a fixed-effects model. The pooled analysis demonstrated that pricking-and-cupping bloodletting therapy significantly decreased the incidence of PHN compared with Western medicine alone (RR = 0.14, 95% CI 0.07–0.27, *p* < 0.001; Figure 9).

Sensitivity analysis demonstrated that the results remained stable after excluding studies with a high risk of bias. Funnel plots hinted at potential existence of publication bias, however, after trim-and-fill correction, the key result persisted. Nevertheless, the funnel plot indicated the presence of publication bias, suggesting possible selective reporting of positive outcomes and a risk of overestimation of the pooled effect. Therefore, clinical interpretation of these results should be undertaken with caution. Details are provided in Supplementary Figures 26–29.

### 3.5 Safety evaluation

Six studies (30, 31, 36, 41, 45, 53) reported adverse reactions. One study (36) contributed two datasets, yielding a total of seven datasets. Overall, 534 participants were included (intervention group: 267 and control group: 267). In four studies (30, 31, 36, 45) (five datasets), no adverse reactions were noted in either the intervention or control groups. In one study (41), one adverse event occurred in the intervention group, accounting for 0.37% of participants. In another study (53), 10 adverse events occurred in the control group, accounting for 3.75% of participants. Due to the limited number of studies, the current evidence is insufficient to draw definitive conclusions regarding safety.

### 3.6 GRADE assessment of certainty of evidence

**Figure 10:**
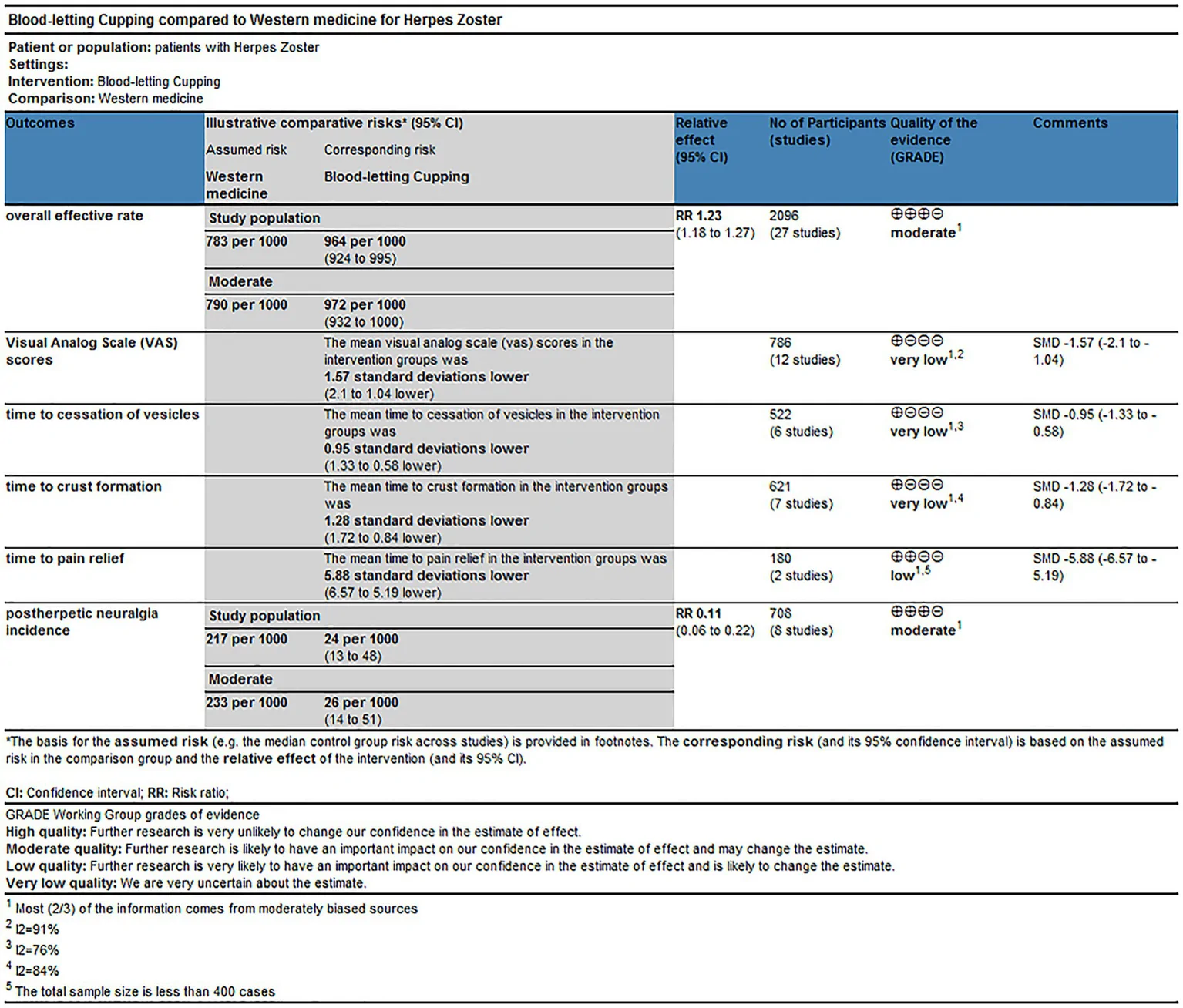
Quality assessment for GRADE evidence.

Using GRADE Profiler (version 3.6), the certainty of evidence was evaluated for the six outcome indicators. Overall, the certainty of evidence was graded as very low, with details visualized in Figure 10.

## 4 Discussion

### 4.1 Effects of pricking-and-cupping bloodletting therapy on pain and skin lesions in HZ

Based on a comprehensive synthesis of the literature, this study indicates that, compared with conventional Western medicine, pricking-and-cupping bloodletting therapy demonstrates a potential beneficial trend in the treatment of HZ. Specifically, it shows promise in reducing Visual Analogue Scale (VAS) pain scores, improving the overall response rate, shortening the time to pain relief, accelerating vesicle cessation and crust formation, and decreasing the incidence of postherpetic neuralgia (PHN). However, according to the GRADE framework, the certainty of the current evidence is very low. Consequently, the observed effects may be overestimated, and these findings should be interpreted with caution. Furthermore, regarding safety, only six studies reported adverse events; therefore, definitive conclusions regarding the safety profile of this therapy remain uncertain.

### 4.2 Mechanisms by which pricking-and-cupping bloodletting therapy alleviates pain and skin lesions in HZ

In accordance with the principle of “removing blood stasis,” bloodletting cupping therapy involves pricking or tapping specific acupoints or collateral vessels on the patient’s body using a plum-blossom needle or three-edged needle to induce cutaneous bleeding, followed by cupping to extract an appropriate amount of blood (20). This method can regulate qi and blood, clear fire, drain heat, resolve stasis, reduce swelling, relieve itching, alleviate pain, open the orifices, and calm the mind (62). In traditional Chinese medicine, HZ falls under the category of “snake-like sores,” which is often attributed to insufficient healthy qi, combined with emotional disturbances and improper diet, leading to the internal generation of damp-heat that attacks the skin surface, resulting in vesicular eruptions and pain. Treatment should focus on clearing heat and removing dampness, as well as unblocking the collaterals and relieving pain. Through bloodletting cupping therapy, damp-heat and pathogenic toxins can be promptly expelled from the body, thereby unblocking the meridians and promoting recovery.

Neuropathic pain induced by HZ typically corresponds to the dermatomal distribution of the affected peripheral nerves. Although its precise pathogenesis remains elusive, it is closely associated with neuroinflammation, cytokine signaling, and peripheral nerve injury. First, regarding neuroinflammation, upon reactivation, the varicella-zoster virus (VZV) synchronously activates satellite glial cells and Schwann cells in the peripheral dorsal root ganglia, as well as microglia and astrocytes in the spinal cord. Proliferating satellite glial cells release pro-inflammatory mediators, while central microglia shift toward the pro-inflammatory M1 phenotype, and astrocytes differentiate into the neurotoxic A1 phenotype. The massive release of inflammatory mediators disrupts both the blood-nerve and blood–brain barriers, establishing a vicious bidirectional cycle between peripheral inflammation and central sensitization. Animal models have confirmed that inhibiting glial activation can significantly alleviate HZ-associated allodynia (63–65), with A1 astrocytes serving as critical mediators for the sustained amplification of central inflammation (66). Second, in terms of cytokine signaling, activated glial cells, neutrophils, and macrophages massively release tumor necrosis factor-alpha (TNF-*α*), interleukin-1β (IL-1β), and interleukin-6 (IL-6). These cytokines form a positive inflammatory feedback cascade via autocrine and paracrine signaling, concurrently upregulating nociceptive neuropeptides such as substance P (SP) and calcitonin gene-related peptide (CGRP). This upregulation increases the excitability of dorsal root neurons, thereby inducing spontaneous pain and hyperalgesia. The massive release of pro-inflammatory mediators by activated macrophages is a crucial trigger for the sustained amplification of inflammation (67). Furthermore, an imbalance in helper T lymphocytes (Th1/Th2) and insufficient secretion of anti-inflammatory cytokines can prolong the pain course, whereas supplementing Th2-related anti-inflammatory signals can alleviate neuropathic sensitization (68). Conversely, excessive activation of regulatory T cells (Tregs) during the acute phase suppresses antiviral immunity, increasing the risk of developing PHN (69). Third, concerning peripheral nerve injury mechanisms, infiltrating immune cells at the lesion site release hydrolytic enzymes and reactive oxygen species (ROS), which directly damage the nerve myelin sheath, leading to demyelination and spontaneous ectopic discharges in injured nerve fibers. Following Schwann cell impairment, the loss of neurotrophic support exacerbates the structural disruption of peripheral nerves (70). Macrophage-secreted cathepsins can sustain the state of allodynia following nerve injury (71), while ROS-mediated myelin degradation serves as a direct trigger for severe pain during the acute phase (72).

Existing clinical studies (23, 24, 73) have demonstrated that pricking-and-cupping bloodletting can upregulate the levels of CD3^+^ and CD4^+^ T lymphocytes and interferon-gamma (IFN-*γ*), while downregulating tumor necrosis factor-alpha (TNF-*α*), interleukin-6 (IL-6), substance P (SP), and calcitonin gene-related peptide (CGRP). By reshaping the Th1/Th2 immune balance, this therapy alleviates acute-phase pain, accelerates the resolution of skin lesions, and reduces the long-term risk of PHN. Integrating these clinical findings with the pathogenic mechanisms of HZ neuropathic pain, a plausible hypothesis can be proposed, that is, the aforementioned clinical benefits may be mediated by the ability of pricking-and-cupping bloodletting to inhibit excessive glial activation, block the inflammatory cascade, and attenuate peripheral nerve demyelination. However, there is currently a lack of direct animal experiments or preclinical studies validating the regulatory effects of this specific pathway. The underlying molecular mechanisms warrant further investigation in future basic research. Therefore, this hypothesis should be regarded strictly as a theoretical deduction at the mechanistic level, rather than a definitive conclusion.

### 4.3 Limitations of the study

Limitations in this study should be noted. First, the exclusive inclusion of Chinese-language publications may introduce linguistic bias. Furthermore, methodological quality assessment of the 31 studies revealed a generally low level of evidence certainty for all outcomes, largely attributable to insufficient rigor in trial design. With specific detailed as follows. Most studies failed to fully implement randomization, including inadequate reporting of random sequence generation and allocation concealment, which may introduce certain selection bias and measurement bias. Due to the inherent characteristics of external traditional Chinese medicine (TCM) interventions, blinding of practitioners and participants could not be achieved according to current evidence-based quality assessment standards; moreover, none of the studies reported blinding of outcome assessors, which may have introduced potential bias. Second, based on the meta-analysis results of this study, pricking-and-cupping bloodletting demonstrates favorable clinical efficacy in alleviating pain and skin lesions associated with HZ. However, assessment using the GRADE framework revealed that the evidence for the primary outcome, VAS score, is of very low certainty. Among the secondary outcomes, the overall response rate and the incidence of PHN are supported by moderate-certainty evidence, time to pain relief by low-certainty evidence, and both time to cessation of vesicles and time to crust formation by very low-certainty evidence. Overall, the very low certainty of evidence for the outcomes in the included studies may compromise the reliability of the study conclusions. Third, regarding the intervention procedures, there were no standardized criteria for the volume of blood extracted, and treatment frequencies varied (e.g., once daily versus once every other day). Furthermore, the lack of uniformity in the Western medicine regimens for the control groups, coupled with varying methodological quality across the included studies, may have affected the consistency and comparability of the therapeutic effects, thereby contributing to heterogeneity. Fourth, the majority of the included studies only reported short-term efficacy and lacked long-term follow-up data, precluding the evaluation of the long-term therapeutic effects of this intervention. Fifth, although sensitivity analyses were conducted after excluding studies with a high risk of bias, and subgroup and meta-regression analyses were performed based on whether pricking-and-cupping was combined with Western medicine and on treatment duration, the sources of heterogeneity remained unidentified. Sixth, publication bias was detected for certain outcomes. Although the application of the trim-and-fill method did not result in a significant reversal of the *p*-value, this still suggests the potential presence of selective reporting or outcome reporting bias favoring positive results. Seventh, all included studies were conducted in China, exclusively enrolling Chinese patients with HZ. The regional specificity in ethnicity, physical constitution, and distribution of underlying diseases may introduce geographic bias. Additionally, as pricking-and-cupping bloodletting is a distinctive traditional Chinese medicine (TCM) therapy with limited adoption in international clinical practice, significant differences in clinical healthcare systems and patient acceptability across countries may introduce cultural treatment bias. Furthermore, Chinese databases are more likely to publish studies with positive outcomes, making it difficult to retrieve and include studies with negative or non-significant results. This may inflate the overall effect size and contribute to publication bias. Consequently, the findings of this study are primarily applicable to the domestic Chinese clinical population and may lack universal international generalizability. Future research should prioritize high-quality, large-sample, multicenter, double-blind randomized controlled trials on a global scale. These trials should incorporate long-term follow-up to observe sustained efficacy and integrate laboratory data as objective outcome measures. Additionally, rigorous clinical trial registration and comprehensive reporting of all outcome indicators are essential. Journals should strive for a balanced publication of both positive and negative findings to mitigate the impact of publication bias on systematic reviews, thereby providing more definitive clinical evidence for the efficacy of pricking-and-cupping bloodletting in treating HZ.

## 5 Conclusion

The pooled data from this meta-analysis suggest a potential beneficial trend of pricking-and-cupping bloodletting therapy in alleviating pain, accelerating skin lesion healing, and reducing the risk of PHN in patients with HZ. However, constrained by multiple limitations—including poor methodological quality of the included studies, extreme clinical heterogeneity, significant publication bias, exclusive reliance on domestic study samples, and a paucity of objective safety data—these findings must be interpreted with extreme caution. Furthermore, given the very low certainty of evidence as assessed by the GRADE framework, pricking-and-cupping bloodletting should not be recommended as a first-line standard treatment for HZ, but rather considered solely as a complementary alternative intervention. Future multicountry, high-quality, and standardized randomized controlled trials are warranted to further validate its true clinical benefits and long-term safety.

## Data Availability

The original contributions presented in the study are included in the article/Supplementary material, further inquiries can be directed to the corresponding author.
