# Supplementary material for: Effects of pricking-and-cupping bloodletting therapy on pain and skin lesions in patients with herpes zoster: a systematic review and meta-analysis
Source: Front Neurol. 2026 Aug 26;17:1806109. doi: 10.3389/fneur.2026.1806109 (PMC13553439; doi:10.3389/fneur.2026.1806109)
Supplement: Supplementary file 1 [file Supplementary_file_1.docx]

Supplemenrary Materials

[Supplementary Table 1. Detailed Search Strategies for Each Database	3](#_Toc236239532)

[Supplementary Table 2. Summary of Specific Procedures, Dosages, and Administration of Interventions and Control Measures Across 31 Studies	5](#_Toc236239533)

[Supplementary Table 3. Criteria for Determining the Overall Response Rate	12](#_Toc236239534)

[Supplementary Figure 1. Regression Analysis Results of VAS by Treatment Course	17](#_Toc236239535)

[Supplementary Figure 2. Meta-Regression Results of VAS Scores by Publication Year	17](#_Toc236239536)

[Supplementary Figure 3. Meta-Regression Results of VAS Scores by Sample Size	17](#_Toc236239537)

[Supplementary Figure 4. Sensitivity Analysis of VAS Scores After Excluding Studies with High Risk of Bias	18](#_Toc236239538)

[Supplementary Figure 5. Egger’s Test Result Plot for VAS Scores	18](#_Toc236239539)

[Supplementary Figure 6. Egger Plot for VAS	19](#_Toc236239540)

[Supplementary Figure 7. Trim-and-Fill Results of VAS Funnel Plot	19](#_Toc236239541)

[Supplementary Figure 8. Trim-and-Fill of VAS Funnel Plot	20](#_Toc236239542)

[Supplementary Figure 9. Sensitivity Analysis of the Overall Response Rate	20](#_Toc236239543)

[Supplementary Figure 10. Egger’s Test Result Plot for the Overall Response Rate	21](#_Toc236239544)

[Supplementary Figure 11. Egger Plot for Overall Response Rate	21](#_Toc236239545)

[Supplementary Figure 13. Meta-regression Analysis of Time to Cessation of Vesicles by Publication Year	22](#_Toc236239546)

[Supplementary Figure 14. Meta-regression Analysis of Time to Cessation of Vesicles by Sample Size	22](#_Toc236239547)

[Supplementary Figure 15. Sensitivity Analysis of Time to Cessation of Vesicles After Excluding Studies with High Risk of Bias	22](#_Toc236239548)

[Supplementary Figure 16. Funnel Plot for Time to Cessation of Vesicles	24](#_Toc236239549)

[Supplementary Figure 17. Trim-and-Fill Results of the Funnel Plot for Time to Cessation of Vesicles	24](#_Toc236239550)

[Supplementary Figure 19. Meta-regression Analysis of Time to Crust Formation by Treatment Course	25](#_Toc236239551)

[Supplementary Figure 20. Meta-regression Analysis of Time to Crust Formation by Publication Year	25](#_Toc236239552)

[Supplementary Figure 21. Meta-regression Analysis of Time to Crust Formation by Sample Size	26](#_Toc236239553)

[Supplementary Figure 22. Sensitivity Analysis of Time to Crust Formation After Excluding Studies with High Risk of Bias	26](#_Toc236239554)

[Supplementary Figure 23. Funnel Plot for Time to Crust Formation	27](#_Toc236239555)

[Supplementary Figure 24. Trim-and-Fill Results of the Funnel Plot for Time to Crust Formation	27](#_Toc236239556)

[Supplementary Figure 25. Trim-and-Fill Analysis of the Funnel Plot for Time to Crust Formation	28](#_Toc236239557)

[Supplementary Figure 26. Sensitivity Analysis of the Incidence of Postherpetic Neuralgia After Excluding Studies with High Risk of Bias	28](#_Toc236239558)

[Supplementary Figure 27. Funnel Plot for the Incidence of Postherpetic Neuralgia	29](#_Toc236239559)

[Supplementary Figure 28. Trim-and-Fill Analysis of the Funnel Plot for the Incidence of Postherpetic Neuralgia (Adjusted)	30](#_Toc236239560)

[Supplementary Figure 29. Trim-and-Fill Analysis of the Funnel Plot for the Incidence of Postherpetic Neuralgia (Unadjusted)	31](#_Toc236239561)

Supplementary Table 1. Detailed Search Strategies for Each Database

1. Overall Chinese search strategy (All databases were searched from database creation to November 2025)

| 研究对象 | 主题词 | 带状疱疹 |
| --- | --- | --- |
|  | 自由词 | 带状疱疹、带状疮疹、蛇窠疮、火丹、蛇串疮、蜘蛛疮、缠腰火丹、火带疮、蛇丹 |
| 干预措施 | 主题词 | 刺络拔罐 |
|  | 自由词 | 刺络拔罐、拔罐放血、刺络放血、放血 |

1.1 Search strategy for CNKI(using advanced search and abstract search)

| 步骤1 | 带状疱疹 + 带状疮疹 + 蛇窠疮 + 火丹 + 蛇串疮 + 蜘蛛疮 + 缠腰火丹 + 火带疮 + 蛇丹 |
| --- | --- |
| 步骤2 | 刺络拔罐 + 拔罐放血 + 刺络放血 + 放血 |
| 步骤3 | 步骤1和步骤2用AND连接 |

1.2 Search strategy for Wanfang (using advanced search and topic search)

| 步骤1 | 带状疱疹 or 带状疮疹 or 蛇窠疮 or 火丹 or 蛇串疮 or 蜘蛛疮 or 缠腰火丹 or火带疮 or 蛇丹 |
| --- | --- |
| 步骤2 | 刺络拔罐 or 拔罐放血 or 刺络放血 or 放血 |
| 步骤3 | 步骤1和步骤2用与连接 |

1.3 Search strategy for VIP (using advanced search and abstract search)

| 步骤1 | 带状疱疹 or 带状疮疹 or 蛇窠疮 or 火丹 or 蛇串疮 or 蜘蛛疮 or 缠腰火丹 or火带疮 or 蛇丹 |
| --- | --- |
| 步骤2 | 刺络拔罐 or 拔罐放血 or 刺络放血 or 放血 |
| 步骤3 | 步骤1和步骤2用与连接 |

1.4 Search strategy for Sinomed (Chinese Biomedical Literature Database) (using advanced search and commonly used fields)

| 步骤1 | ("带状疱疹"[常用字段:智能] OR "带状疮疹"[常用字段:智能] OR "蛇窠疮"[常用字段:智能] OR "火丹"[常用字段:智能] OR "蛇串疮"[常用字段:智能] OR "蜘蛛疮"[常用字段:智能] OR "缠腰火丹"[常用字段:智能] OR "火带疮"[常用字段:智能] OR "蛇丹"[常用字段:智能]) |
| --- | --- |
| 步骤2 | "刺络拔罐"[常用字段:智能] OR "拔罐放血"[常用字段:智能] OR "刺络放血"[常用字段] OR "放血" |
| 步骤3 | 步骤1和步骤2用AND连接 |

2. Search strategies for English database

2.1 Search strategy for PubMed

| 1 | Herpes Zoster[MeSH Terms] |
| --- | --- |
| 2 | 'disseminated herpes zoster'[Title/Abstract] OR 'herpes zona'[Title/Abstract] OR 'herpes zoster disease'[Title/Abstract] OR 'herpes zoster infection'[Title/Abstract] OR 'herpes zoster neuralgia'[Title/Abstract] OR 'herpes zoster paralysis'[Title/Abstract] OR 'shingles'[Title/Abstract] OR 'varicella zoster infection'[Title/Abstract] OR 'varicella zoster viral infection'[Title/Abstract] OR 'Varicella zoster virus infection'[Title/Abstract] OR 'Varicellovirus infection'[Title/Abstract] OR 'VZV infection'[Title/Abstract] OR 'zoster'[Title/Abstract] OR 'herpes zoster'[Title/Abstract] OR 'Zona'[Title/Abstract] |
| 3 | (Herpes Zoster[MeSH Terms]) OR ('disseminated herpes zoster'[Title/Abstract] OR 'herpes zona'[Title/Abstract] OR 'herpes zoster disease'[Title/Abstract] OR 'herpes zoster infection'[Title/Abstract] OR 'herpes zoster neuralgia'[Title/Abstract] OR 'herpes zoster paralysis'[Title/Abstract] OR 'shingles'[Title/Abstract] OR 'varicella zoster infection'[Title/Abstract] OR 'varicella zoster viral infection'[Title/Abstract] OR 'Varicella zoster virus infection'[Title/Abstract] OR 'Varicellovirus infection'[Title/Abstract] OR 'VZV infection'[Title/Abstract] OR 'zoster'[Title/Abstract] OR 'herpes zoster'[Title/Abstract] OR 'Zona'[Title/Abstract]) |
| 4 | bloodletting[MeSH Terms] |
| 5 | 'Bloodletting'[Title/Abstract] OR 'pricking blood treatment'[Title/Abstract] OR 'pricking-cupping'[Title/Abstract] OR 'cupping'[Title/Abstract] |
| 6 | (bloodletting[MeSH Terms]) OR ('Bloodletting'[Title/Abstract] OR 'pricking blood treatment'[Title/Abstract] OR 'pricking-cupping'[Title/Abstract] OR 'cupping'[Title/Abstract]) |
| 7 | ((Herpes Zoster[MeSH Terms]) OR ('disseminated herpes zoster'[Title/Abstract] OR 'herpes zona'[Title/Abstract] OR 'herpes zoster disease'[Title/Abstract] OR 'herpes zoster infection'[Title/Abstract] OR 'herpes zoster neuralgia'[Title/Abstract] OR 'herpes zoster paralysis'[Title/Abstract] OR 'shingles'[Title/Abstract] OR 'varicella zoster infection'[Title/Abstract] OR 'varicella zoster viral infection'[Title/Abstract] OR 'Varicella zoster virus infection'[Title/Abstract] OR 'Varicellovirus infection'[Title/Abstract] OR 'VZV infection'[Title/Abstract] OR 'zoster'[Title/Abstract] OR 'herpes zoster'[Title/Abstract] OR 'Zona'[Title/Abstract])) AND ((bloodletting[MeSH Terms]) OR ('Bloodletting'[Title/Abstract] OR 'pricking blood treatment'[Title/Abstract] OR 'pricking-cupping'[Title/Abstract] OR 'cupping'[Title/Abstract])) |

2.2 Search strategy for EMbase

| 1 | #1 'herpes Zoster'/exp |
| --- | --- |
| 2 | #2 'disseminated herpes zoster':ti,ab,kw OR 'herpes zona':ti,ab,kw OR 'herpes zoster disease':ti,ab,kw OR 'herpes zoster infection':ti,ab,kw OR 'herpes zoster neuralgia':ti,ab,kw OR 'herpes zoster paralysis':ti,ab,kw OR 'shingles':ti,ab,kw OR 'varicella zoster infection':ti,ab,kw OR 'varicella zoster viral infection':ti,ab,kw OR 'Varicella zoster virus infection':ti,ab,kw OR 'Varicellovirus infection':ti,ab,kw OR 'VZV infection':ti,ab,kw OR 'zoster':ti,ab,kw OR 'herpes zoster':ti,ab,kw OR 'Zona':ti,ab,kw |
| 3 | #3 #1 OR #2 |
| 4 | #4 'bloodletting'/exp |
| 5 | #5 'Bloodletting':ti,ab,kw OR 'pricking blood treatment':ti,ab,kw OR 'pricking-cupping':ti,ab,kw OR 'cupping':ti,ab,kw |
| 6 | #6 #4 OR #5 |
| 7 | #7 #3 AND #6 |

2.3 Search strategy for Cochrane Library

| 1 | #1 MeSH descriptor: [ Herpes Zoster] explode all trees |
| --- | --- |
| 2 | #2(disseminated herpes zoster):ti,ab,kw OR (herpes zona):ti,ab,kw OR (herpes zoster disease):ti,ab,kw OR (herpes zoster infection) :ti,ab,kw OR (herpes zoster neuralgia):ti,ab,kw OR (herpes zoster paralysis):ti,ab,kw OR (shingles):ti,ab,kw OR (varicella zoster infection):ti,ab,kw OR (varicella zoster viral infection):ti,ab,kw OR (Varicella zoster virus infection):ti,ab,kw OR (Varicellovirus infection):ti,ab,kw OR (VZV infection):ti,ab,kw OR (zoster) OR (herpes zoster):ti,ab,kw OR (Zona):ti,ab,kw |
| 3 | #3 #1 OR #2 |
| 4 | #4 MeSH descriptor: [ Bloodletting] explode all trees |
| 5 | #5 (Bloodletting):ti,ab,kw OR (pricking blood treatment):ti,ab,kw OR (pricking-cupping):ti,ab,kw OR (cupping):ti,ab,kw |
| 6 | #6 #4 OR #5 |
| 7 | #7 #3 AND #6 |

2.4 Search strategy for Web of Science

| **1** | **#1TS=('disseminated herpes zoster' OR 'herpes zona' OR 'herpes zoster disease' OR 'herpes zoster infection' OR 'herpes zoster neuralgia' OR 'herpes zoster paralysis' OR 'shingles' OR 'varicella zoster infection' OR 'varicella zoster viral infection' OR 'Varicella zoster virus infection' OR 'Varicellovirus infection' OR 'VZV infection' OR 'zoster' OR 'herpes zoster' OR 'Zona')** |
| --- | --- |
| **2** | #2**TS=('Bloodletting' OR 'pricking blood treatment' OR 'pricking-cupping' OR 'cupping')59266** |
| **3** | **#1 AND #2** |

Supplementary Table 2. Summary of Specific Procedures, Dosages, and Administration of Interventions and Control Measures Across 31 Studies

| Author | Pricking-and-Cupping Bloodletting (Alone or Combined) | Conventional Western Medicine |
| --- | --- | --- |
| Liu Shu [30] | Pricking-and-cupping bloodletting + Western medicine  Location: Jiaji points corresponding to the spinal segments of the painful area, and local Ashi points (2–3 points with the most pronounced tenderness on palpation within the herpes cluster area).  Procedure: The patient was placed in an appropriate position to fully expose the affected skin. After routine iodophor disinfection, a disposable syringe needle (specification: 0.7 mm) was used for rapid pricking, with 3–5 pricks per point until the blisters ruptured or slight skin bleeding occurred, with a spacing of 10 mm between each prick. Blood oozing points were observed on the affected skin. A glass cupping jar was then immediately applied to the oozing points, and upon observing blood drainage, the cup was retained for 5–10 min. After cup removal, the congested blood was wiped away with a dry cotton ball, and the skin was disinfected with iodophor to prevent infection. Frequency: once daily.  Duration: 14 days. | Gabapentin capsules (0.1 g × 50 capsules)  Dosage: 3 capsules per dose, orally, three times daily.  Mecobalamin tablets (0.5 mg × 40 tablets)  Dosage: 3 tablets per dose, orally, three times daily.  Duration: 14 days |
| Li Liang [31] | Pricking-and-cupping bloodletting + Western medicine  Procedure: The herpes lesions and a three-edged needle were disinfected with iodophor. The area around the herpes lesions was pricked 3–4 times until blood droplets appeared, which were then wiped away and compressed for hemostasis. After hemostasis, cupping therapy was performed with the cup retained for 3–5 min. Following cupping, the area was disinfected again and covered with sterile gauze. Frequency: once every 3 days, for a total of 4 treatments.  Duration: 12 days. | Antiviral drugs, intramuscular vitamin B1, and analgesic drugs.  Duration: 12 days. |
| Gai Xingxing [32] | Pricking-and-cupping bloodletting  Procedure: The herpes zoster area was locally disinfected (medical iodine). A sterilized three-edged needle was used to prick the herpes vesicle area 2–3 times. After bead-like blood droplets emerged, cupping was applied at the pricking sites. Each session involved pricking 2–3 vesicles. Seven sessions constituted one treatment course, and two courses were administered. | Acyclovir tablets (specification: 0.1 g × 30 tablets)  Dosage: 0.2 g per dose, 3 times daily, orally.  Mecobalamin tablets (specification: 0.5 mg)  Dosage: 0.5 mg per dose, 3 times daily, orally.  Duration: 14 days. |
| Wang Mi [33] | Pricking-and-cupping bloodletting + Western medicine  Procedure: The exposed affected area was disinfected point by point. After confirming the Ashi points, a plum-blossom needle was used for tapping. Upon observing blood exudation from the local skin, a sterilized glass cupping jar was applied to the affected skin area with suction. The cup was retained for 8 min, and after 3 mL of blood was drained, the cup was removed and the area was disinfected. Frequency: once daily.  Duration: 7 days. | Acyclovir (specification: 0.1 g × 24 tablets)  Dosage: 0.8 g per dose, 5 times daily, orally.  Duration: 7 days. |
| Li Hui [34] | Pricking-and-cupping bloodletting + Western medicine  Procedure: The local lesion area (herpes-affected area or nerve distribution pain area) and its surroundings were routinely disinfected with Anerdian (iodine disinfectant). A disposable 1 mL syringe needle was used to first prick the Ashi points around the lesion, followed by scattered pricking of the lesion area itself to induce bleeding, with a pricking depth of 1–2 mm. Based on the size of the lesion area, appropriate sizes and quantities of fire cups were selected, and the fire-flash method was used to apply cupping over the pricked bleeding sites. For areas unsuitable for fire cups, appropriately sized suction cups could be used as a substitute. The volume of blood drained was 3–5 mL, and each cup was retained for 3–5 min. Frequency: once daily. Duration: 7 days. | Acyclovir sodium injection  Dosage: 0.5 g added to intravenous fluid, administered once every 8 hours. Mecobalamin  Dosage: 0.5 mg, 3 times daily, orally.  Duration: 7 days. |
| Cao Lei [35] | Pricking-and-cupping bloodletting + Western medicine  Procedure: The skin was first disinfected with 75% alcohol cotton balls. A plum-blossom needle was then used to rapidly prick the herpes lesions until the skin appeared flushed with slight blood exudation. A fire cup was immediately applied to the pricked area. After suction was established, the cup was retained for 5–10 min, with the duration determined by careful observation of the amount of bleeding. Following cup removal, the affected skin was wiped and disinfected with 75% alcohol cotton swabs, and sterile gauze was applied externally. Frequency: once every 7 days. Duration: 30 days. | Famciclovir  Dosage: 0.125 g, 3 times daily, orally for 7 days.  Mecobalamin tablets  Dosage: 0.5 mg, 3 times daily, orally for 30 days.  Duration: 30 days. |
| Zhang Xiaogang [36] | Pricking-and-cupping bloodletting + Western medicine  Procedure: The local skin of the herpes zoster lesion was routinely disinfected. Then, 8–10 Ashi points around the herpes lesion were selected. The three-edged needle was held by the handle with the tip directed toward the herpes lesion, and pricking was performed to a depth of approximately 2 mm until slight local blood exudation was observed. An appropriately sized cupping jar was then selected and applied to the pricked area using the fire-flash method. The cup was removed after 5–10 min, with a blood drainage volume of 5–10 mL. After cup removal, the blood stains were wiped clean with cotton balls, and the area was disinfected with iodophor. Frequency: once every other day.  Duration: 7 days. | Acyclovir tablets  Dosage: 0.4 g, 3 times daily, orally on an empty stomach before meals.  Mecobalamin tablets  Dosage: 0.5 mg, 3 times daily, orally on an empty stomach before meals. Duration: 7 days. |
| Zhang Xiaogang [36] | Pricking-and-cupping bloodletting  Procedure: The local skin of the herpes zoster lesion was routinely disinfected. Then, 8–10 Ashi points around the herpes lesion were selected. The three-edged needle was held by the handle with the tip directed toward the herpes lesion, and pricking was performed to a depth of approximately 2 mm until slight local blood exudation was observed. An appropriately sized cupping jar was then selected and applied to the pricked area using the fire-flash method. The cup was removed after 5–10 min, with a blood drainage volume of 5–10 mL. After cup removal, the blood stains were wiped clean with cotton balls, and the area was disinfected with iodophor. Frequency: once every other day.  Duration: 7 days. | Acyclovir tablets  Dosage: 0.4 g, 3 times daily, orally on an empty stomach before meals.  Mecobalamin tablets  Dosage: 0.5 mg, 3 times daily, orally on an empty stomach before meals. Duration: 7 days. |
| Shi Jiajun [37] | Pricking-and-cupping bloodletting + Western medicine  Procedure: The herpes-affected skin was fully exposed. Ashi points and the local lesion area were selected. After iodophor disinfection, the iodine was removed with 75% alcohol. A plum-blossom needle was held by the handle and used for tapping-pricking, with the force adjusted to induce slight skin bleeding. After tapping, cupping was applied to the tapped areas, with 3 cups applied per session. The size of the glass cup was determined based on the size of the lesion area. The cup was retained for 5 min, and the blood drainage volume was controlled at approximately 3 mL. Upon cup removal, the contaminated blood on the local skin was wiped clean with a dry sterile cotton ball, followed by wiping of blood residue with 75% alcohol. The patient was instructed to keep the treated skin area dry. Frequency: once every other day, for a total of 7 treatments.  Duration: 14 days. | Valacyclovir hydrochloride tablets  Dosage: 300 mg per dose, 2 times daily, orally.  Mecobalamin tablets  Dosage: 0.5 mg per dose, 3 times daily, orally.  Duration: 14 days. |
| Meng Zhiyang [38] | Pricking-and-cupping bloodletting + Western medicine  Procedure: The lesion area was fully exposed. After local disinfection with 75% medical alcohol cotton balls, a sterile plum-blossom needle was used to evenly tap-prick the herpes lesions, causing the blisters to rupture and bleed. Cupping was then applied to the tapped areas using the fire-flash method, with the cup retained for 5–10 min to drain approximately 10 mL of blood. The blood stains were wiped clean with sterile gauze, followed by routine disinfection with 75% medical alcohol cotton balls. After the first treatment, the herpes lesions turned black and crusted. If new herpes lesions appeared the next day, the treatment was repeated once, with an additional treatment on day 4. If no new herpes lesions appeared, plum-blossom needle tapping and cupping were performed on the areas with severe pain on day 3 and day 5. Each patient received a total of 3 treatments.  Duration: 7 days. | Acyclovir tablets (specification: 0.2 g per tablet)  Dosage: 2 tablets per dose, 5 times daily, orally.  Prednisone acetate tablets (specification: 5 mg per tablet) Dosage: 6 tablets per dose, once daily, orally.  Mecobalamin tablets (specification: 0.5 mg per tablet)  Dosage: 1 tablet per dose, 3 times daily, orally.  Pregabalin capsules (specification: 75 mg per capsule)  Dosage: 1 capsule per dose, once daily, orally.  Vitamin B1 (specification: 10 mg per tablet)  Dosage: 1 tablet per dose, 3 times daily, orally.  Duration: 7 days. |
| Wu Yufeng [39] | Pricking-and-cupping bloodletting + Western medicine  Procedure: The skin around the lesion area was routinely disinfected 3 times with iodophor cotton swabs. The skin was stretched with the left hand, while the right hand held a three-edged needle for rapid pricking and picking of local Ashi points, to a depth of 1–3 mm, with an interval of approximately 1.0 cm between each prick. Within the area of each cup, 5–7 pricks were made. Cupping was then rapidly applied using the fire-flash method, with 3–7 cups per session, and the cups were retained for 15 min. After cup removal, the pricked area was treated with iodophor disinfection and sterile dry cotton swabs. Frequency: once every 2 days.  Duration: 15 days. | Antibiotics and vitamin medications were administered symptomatically as needed.  Ganciclovir tablets  Dosage: 0.5 g, 3 times daily, orally.  Duration: 15 days. |
| Wang Gang [40] | Pricking-and-cupping bloodletting  Cupping and bloodletting acupoints: The herpes zoster lesion area was selected. Procedure: ① The herpes lesion was locally disinfected with iodophor, and the iodine was removed with dry cotton. ② A No. 5 glass cup was applied to the herpes lesion (covering all herpes lesions) for cupping therapy, and the cup was removed after 10 min. ③ After cup removal, a plum-blossom needle (or three-edged needle, selected according to the condition) was used for pricking, followed by another cupping treatment, with the cup removed after 5–10 min. ④ After cup removal, the pricked area was routinely disinfected without any bandaging or gauze application. ⑤ Cupping was performed daily, with pricking performed once every 2 days.  Duration: 7 days. | Acyclovir chewable tablets (specification: 0.4 g/tablet)  Dosage: 2 tablets per dose, 3 times daily, orally.  Mecobalamin tablets (specification: 0.5 mg/tablet)  Dosage: 1 tablet per dose, 3 times daily, orally.  Duration: 7 days. |
| Cui Jing [41] | Pricking-and-cupping bloodletting + Western medicine  Procedure: After routine disinfection of the herpes lesion area, a plum-blossom needle was used to puncture the blisters, followed by cupping treatment. The procedure was completed once the blister fluid was suctioned out. After 3 days of treatment, a 1-day interval was taken before starting the second treatment cycle.  Duration: 7 days. | Famciclovir dispersible tablets  Dosage: 0.25 g per dose, 3 times daily, orally.  Recombinant human interferon α1b for injection  Dosage: 30 μg per dose, once daily, intramuscular injection.  Duration: 7 days. |
| Jiang Guilin [42] | Pricking-and-cupping bloodletting + Western medicine  The herpes lesion area was fully exposed and cleaned with normal saline cotton balls. If the blister surface was broken, the area was first disinfected with iodophor cotton balls, followed by alcohol cotton ball disinfection of the surrounding skin, and then allowed to dry. The skin around the herpes lesions was tapped with a plum-blossom needle until slight bleeding occurred. An appropriately sized fire cup was then selected for cupping therapy, with the cup retained for 10–15 min. Finally, the skin surface was wiped clean with sterile dry cotton balls.  Duration: 6 days constituted one treatment course, with rest on weekends, for a total of 2 courses. | Valacyclovir hydrochloride dispersible tablets (0.15 g/tablet)  Dosage: 0.3 g per dose, 2 times daily, orally.  Mecobalamin tablets (0.5 mg/tablet) Dosage: 0.5 mg per dose, 3 times daily, orally.  Vitamin B1 (10 mg/tablet)  Dosage: 10 mg per dose, 3 times daily, orally.  Duration: 15 days. |
| Cai Xiaoce [43] | Pricking-and-cupping bloodletting  Procedure: The herpes zoster area was disinfected with iodine tincture, and the iodine was removed with 75% alcohol. A No. 7 infusion set needle was used to prick 3–5 times per 1 cm² of the lesion area. The fire-flash method was immediately applied to the pricked sites for cupping, with the cups retained for 3–5 min to drain 5–10 mL of blood. After cup removal, the area was disinfected again. Frequency: once every 5 days. Ten days constituted one treatment course, and two consecutive courses were administered.  Total duration: 20 days. | Vidarabine (intravenous)  Dosage: 0.4 g per dose, once daily, intravenous infusion.  Vitamin B12  Dosage: 0.5 mg per dose, once daily, intramuscular injection.  Vitamin B1  Dosage: 100 mg per dose, 2 times daily, orally.  Mecobalamin  Dosage: 0.5 mg per dose, 2 times daily, orally.  Acyclovir ointment  Usage: topical application.  Ten days constituted one treatment course, and two consecutive courses were administered.  Total duration: 20 days. |
| Pang Jian'en [44] | Pricking-and-cupping bloodletting + Western medicine  Procedure: The herpes zoster area was first routinely disinfected, and the blister fluid was squeezed out. A plum-blossom needle was then used to tap-prick the herpes area until local skin bleeding occurred. Cupping was applied to the bleeding sites for approximately 15 min. If the herpes lesions were located on the patient's face, flash cupping was used instead. After cup removal, the herpes area was wiped with iodophor solution. After the first bloodletting cupping treatment, the procedure was repeated once every other day. <br> One week constituted one treatment course, and two consecutive courses were administered.  Total duration: 14 days. | Acyclovir sustained-release tablets  Dosage: 0.1 g per dose, 4 times daily, orally.  Acyclovir ointment  Usage: topical application, 3 times daily.  Cobamamide  Dosage: 1.5 mg per dose, once daily, intramuscular injection.  Seven consecutive days constituted one treatment course, and two consecutive courses were administered.  Total duration: 14 days. |
| Li Zhiquan [45] | Pricking-and-cupping bloodletting  Procedure: The affected skin was fully exposed. After routine disinfection, the lesion and painful areas were tap-pricked using a stable, accurate, and rapid technique with gradually increasing force, until slight epidermal oozing of blood was observed. A medium-sized glass fire cup was then applied to the oozing area and retained for 1–2 min to induce slight epidermal bleeding. After cup removal, the stagnant blood was wiped away with sterile gauze, and iodophor was applied. Frequency: once every 3 days.  Ten days constituted one treatment course. | Acyclovir  Dosage: 0.2 g per dose, 3 times daily, orally.  Mecobalamin  Dosage: 0.5 mg per dose, 3 times daily, orally.  Ten days constituted one treatment course. |
| Xing Yukui [46] | Pricking-and-cupping bloodletting  Procedure: A cutaneous needle was used to tap-prick the lesion area, with the intensity determined by the appearance of marked skin flushing and slight bleeding. Fire-flash cupping was applied to the pricked sites. The number of cups and the volume of blood drainage were determined based on the patient's individual condition. During cup retention, changes in skin color and bleeding status were carefully monitored. After cup removal, the contaminated blood was cleaned with sterile dry cotton balls. Frequency: once daily.  Seven days constituted one treatment course, and two courses were administered. Total duration: 14 days. | Foscarnet sodium chloride  Dosage: 3 g once daily, intravenous infusion.  Valacyclovir capsules  Dosage: 0.15 g twice daily, orally. Seven days constituted one treatment course, and two courses were administered.  Total duration: 14 days |
| Bai Meng [47] | Pricking-and-cupping bloodletting + Western medicine  Procedure: The patient's Ashi points were selected. Vacuum cupping was first applied to the pain area for 15 min. The pain area was then disinfected, and a three-edged needle was used to prick 5–10 points to a depth of 1–2 cun. Cupping was then reapplied to the area for 5 min. If the patient's clinical manifestations did not completely resolve after the first treatment session, the pain area was treated again. The blood drainage volume was determined based on the patient's age, general physical condition, pain perception, and blood color, and was generally set at 15–20 mL. The cup was positioned to completely cover the pain area. After bloodletting was completed, the pain area was disinfected, cupped, and bled again, followed by external application of gauze. Frequency: once every other day.  Duration: 10 days. | Acyclovir ointment  Usage: topical application to the affected area, 3 times daily.  Acyclovir tablets  Dosage: 0.4 g per dose, 5 times daily, orally.  Duration: 10 days. |
| Wei Fan [48] | Pricking-and-cupping bloodletting + Western medicine  Procedure: The herpes lesion area and surrounding skin were routinely disinfected. First, the blister fluid was drained by tap-pricking the herpes area with a plum-blossom needle until local skin bleeding was achieved. Cupping was then applied to the tap-pricked area for 10–15 min. For facial lesions, flash cupping was primarily used, and prolonged cup retention was avoided. After cup removal, the area was wiped clean with iodophor. Frequency: once daily or once every other day, with the treatment duration determined by pain relief and herpes crusting. Treatment was administered daily from day 1 to day 7, and once every other day from day 8 to day 21. The size of the tap-pricking area and the number of cups were determined based on the extent of the lesion. A total of 21 days of treatment was administered. For patients with symptomatic improvement during treatment, the frequency of treatment could be reduced; for those with severe symptoms, daily treatment could be administered.  Total duration: 21 days. | Acyclovir sustained-release tablets  Dosage: 0.1 g per dose, 4 times daily, orally.  Acyclovir ointment  Usage: topical application, 3 times daily.  Cobamamide  Dosage: 1.5 mg per dose, once daily, intramuscular injection.  Total duration: 21 days. |
| Tong Laisheng [49] | Pricking-and-cupping bloodletting + Western medicine  Procedure: Ashi points were selected. Vacuum cupping was applied to the pain area for 15 min, after which the area was disinfected. A three-edged needle was used to prick 5–10 points to a depth of 1–2 cun, followed by another cupping session for 5 min. If symptoms did not completely resolve after the first treatment, a second treatment was administered based on the pain area. The blood drainage volume was 15–20 mL, and the cup was positioned to cover the affected area. The blood drainage volume was determined based on the patient's age, constitution, pain severity, and blood color. After bloodletting was completed, the affected area was disinfected, cupped, and bled again, followed by external application of gauze. Frequency: once every other day.  Duration: 10 days. | Acyclovir ointment  Usage: topical application to the affected area, 3 times daily.  Acyclovir tablets (oral)  Dosage: 0.4 g per dose, 5 times daily, orally.  Duration: 10 days. |
| Li Hu [50] | Pricking-and-cupping bloodletting + Western medicine  Procedure: The affected area was fully exposed and routinely disinfected with iodophor. The operator held the handle of a seven-star needle with the right hand, with the index finger pressing on the needle handle. Using wrist force, the herpes lesions and surrounding skin, or the skin along the meridian course, were rapidly tap-pricked until slight bleeding was observed. For large blisters, a three-edged needle was used for rapid pricking, and the blister fluid or blood was wiped away with a dry cotton ball. Based on the size of the herpes and pricking area, an appropriately sized glass cup was selected and quickly applied using the fire-flash method. The cup was retained for 10 min. After cup removal, the blood stains were cleaned with a sterile cotton ball, and the area was disinfected with iodophor. For unruptured or recurrent herpes lesions, the procedure could be repeated. This method was discontinued after herpes crusting. Treatment was administered once every other day, with 3 treatments typically constituting one treatment course.  Total duration: 10 days. | Famciclovir  Dosage: 250 mg, 3 times daily, orally.  Mecobalamin  Dosage: 0.5 mg, 3 times daily, orally. Duration: 10 days. |
| Chen Wei [51] | Pricking-and-cupping bloodletting  Procedure: The lesion area and surrounding skin were routinely disinfected with iodine tincture and alcohol. A plum-blossom needle was then used to tap-prick the lesion area, moving from the periphery to the center, repeatedly and rapidly, to a depth sufficient to induce slight skin bleeding. Immediately after bleeding was achieved, cupping was applied to the affected area, with the cup positioned to cover all lesions and bloodletting sites. The cup was retained for 10–15 min, with the goal of drawing out the exudate and stagnant blood from the herpes lesions and inducing local skin congestion to a red or purplish appearance. The affected area was then cleaned with an alcohol cotton ball. Treatment was administered once every other day, and after 2 treatments, a 2-day rest was taken. Five days constituted one treatment course, for a total of 2 courses.  Total duration: 10 days. | Valacyclovir hydrochloride tablets (specification: 0.15 g)  Dosage: 0.3 g per dose, 2 times daily, orally on an empty stomach before meals.  Five days constituted one treatment course, for a total of 2 courses.  Total duration: 10 days. |
| Gao Jianzhong [52] | Pricking-and-cupping bloodletting  Procedure: After routine disinfection, a three-edged needle was used to prick the herpes lesions. One needle was quickly inserted on each side, 5 mm from the herpes margin. Cupping was immediately applied locally, with a blood drainage volume of 2–5 mL, and the cup was retained for 10–15 min. Treatment was administered once every other day, with 5 sessions constituting one treatment course.  Duration: 10 days. | Acyclovir  Dosage: 0.8 g per dose, 3 times daily, orally.  Duration: 10 days. |
| Zhao Bohua [53] | Pricking-and-cupping bloodletting  Procedure: An appropriate position was selected based on the lesion location. After strict disinfection of the affected skin, a plum-blossom needle was used to gently and evenly tap-prick centripetally from one end of the post-herpetic pain area along the margin of the skin lesion at Ashi points, surrounding the lesion. After encircling the lesion, the herpes and painful areas were tap-pricked with greater force to induce punctate oozing bleeding. Cupping was then immediately applied to the tap-pricked area, with the cup retained for 5 min to suction out the serous exudate and oozing blood. After cup removal, the area was locally disinfected. Treatment was administered once every other day. Seven sessions constituted one treatment course, for a total of 2 courses.  Duration: 14 days. | Ribavirin tablets  Dosage: 0.3 g per dose, 3 times daily, orally.  Carbamazepine  Dosage: 0.1 g per dose, 3 times daily, orally.  Vitamin B1  Dosage: 10 mg per dose, 3 times daily, orally.  Mecobalamin  Dosage: 0.5 mg per dose, 3 times daily, orally.  Prednisone acetate  Dosage: 10 mg per dose, 3 times daily, orally.  Fourteen days constituted one treatment course.  Duration: 14 days. |
| Pan Dong [54] | Pricking-and-cupping bloodletting  Procedure: The skin around the concentrated herpes lesions was disinfected with 75% alcohol cotton balls. A three-edged needle was used to scatter-prick the disinfected area. Several fire cups with a diameter of 5 cm were applied over the area for 8 min. After treatment, the blood stains were dried with sterile dry cotton balls, and the skin was covered with sterile gauze. Treatment was administered once daily.  Duration: 14 days. | Famciclovir (specification: 0.125 g)  Dosage: 0.25 g per dose, 3 times daily, orally.  Duration: 14 days. |
| Liu Haiying [55] | Pricking-and-cupping bloodletting  Procedure: After routine disinfection of the local lesion area, a three-edged needle was used to prick the herpes lesions and surrounding skin until the blisters were ruptured, fluid drained, and the surrounding skin became congested. Cupping was then applied to the pricked sites for 10–15 min, with a blood drainage volume of 5–8 mL. Treatment was administered once daily, with 7 days constituting one treatment course.  Duration: 7 days. | Acyclovir  Dosage: 0.2 g per dose, 3 times daily, orally.  Indomethacin  Dosage: 25 mg per dose, 3 times daily, orally after meals.  Compound vitamin B  Dosage: 3 tablets per dose, 3 times daily, orally.  Seven days constituted one treatment course.  Duration: 7 days. |
| Shao Suju [56] | Pricking-and-cupping bloodletting  Procedure: The herpes area and surrounding skin were routinely disinfected. A cutaneous needle was first used to tap-prick the herpes area until the blister fluid drained and local skin bleeding was achieved. Cupping was then applied to the tap-pricked area for 10–15 min. After cup removal, the area was wiped clean with a sterile cotton ball and covered with a single layer of sterile gauze. Treatment was administered once daily, with 7 sessions constituting one treatment course.  Duration: 7 days. | Acyclovir sustained-release tablets  Dosage: 0.8 g per dose, 3 times daily, orally.  Phthiobuzone liniment  Usage: topical application to the affected area, 3 times daily.  Duration: 7 days. |
| Song Suyan [57] | Pricking-and-cupping bloodletting  Procedure: The herpes lesion area was disinfected with medical iodine. A three-edged needle was used to prick 2–3 times around the herpes blisters until bead-like blood droplets appeared. Cupping was then applied to the pricked bleeding sites. Each session involved pricking 2–3 small blisters. Seven sessions constituted one treatment course.  Duration: 7 days. | Acyclovir injection  Dosage: 5 mg/kg, intravenous infusion, 3 times daily (once every 8 hours).  Seven days constituted one treatment course.  Duration: 7 days. |
| Wu Huajun [58] | Pricking-and-cupping bloodletting  Procedure: One to two sites were selected from the initial herpes lesion, the last appearing herpes lesion, and the area with the most herpes lesions. After routine disinfection, each site was tap-pricked 2–3 times with a sterile plum-blossom needle until the blisters were ruptured (different sites were pricked daily). A sterile glass cup was applied to the tap-pricked sites using the fire-flash method and retained for 1–3 min, with 3–5 mL of blood and stagnant blood suctioned out. The area was then wiped clean with sterile cotton, disinfected with iodophor, followed by 75% alcohol.  Duration: 10 days. | Recombinant human interferon α1b  Dosage: 50 μg dissolved in 1 mL of sterile water for injection, intramuscular injection, once daily.  Acyclovir injection  Usage: topical application, 3–4 times daily.  Ten sessions constituted one treatment course.  Duration: 10 days. |
| Zhang Yalan [59] | Pricking-and-cupping bloodletting  Procedure: The affected area was disinfected with 75% alcohol. A three-edged needle or thick filiform needle was used for encircling pricking around the herpes clusters. If the area was large, it could be treated in sections. The skin was pricked with a stable, accurate, and rapid technique to a depth of 0.5–1 fen, with each point spaced approximately 0.2 cm apart. After all herpes blisters were pricked, cupping was rapidly applied using the fire-flash method, with 5–10 mL of blood drained per cup as appropriate. The cups were removed once the herpes color gradually turned dark red, and the area was wiped clean with a sterile dry cotton ball. Treatment was administered once every other day, for a total of 2–4 sessions.  Duration: 7 days. | Acyclovir  Dosage: 0.2 g per dose, 5 times daily, orally.  Vitamin B1  Dosage: 20 mg per dose, 3 times daily, orally.  Ibuprofen  Dosage: 300 mg per dose, 2 times daily, orally.  Calamine lotion  Usage: topical application to the affected area.  Continuous treatment for 1 week.  Duration: 7 days. |
| Xu Lan [60] | Pricking-and-cupping bloodletting + Western medicine  Procedure: The skin lesion area was routinely disinfected with 75% alcohol. A sterilized three-edged needle was used to prick each blister one by one, allowing the blister fluid to drain. If there were no blisters, the erythematous and infected painful skin was pricked to induce bleeding. Cupping was then applied, and after 15 min, the cup was removed, and the suctioned blister fluid or stagnant blood was wiped away. Seven days constituted one treatment course.  Duration: 7 days. | Acyclovir for injection  Dosage: 0.5 g, dissolved in 250 mL of 5% glucose injection, intravenous infusion, 2 times daily.  Acyclovir cream  Usage: topical application to the affected area.  Seven days constituted one treatment course.  Duration: 7 days. |

Supplementary Table 3. Criteria for Determining the Overall Response Rate

| Author | Criteria for Efficacy Evaluation |
| --- | --- |
| Liu Shu [30] | The efficacy evaluation criteria were formulated with reference to the *Guiding Principles of Clinical Research on New Traditional Chinese Medicine Drugs* and the *TCM Diagnostic and Therapeutic Criteria for Diseases and Syndromes*.  Therapeutic index = [(Pre-treatment VAS score − Post-treatment VAS score) / Pre-treatment VAS score] × 100%.  Clinical cure: Therapeutic index ≥ 95%.  Markedly effective: 60% ≤ Therapeutic index < 95%.  Effective: 30% ≤ Therapeutic index < 60%.  Ineffective: Therapeutic index < 30%.  Overall response rate = (Number of cases with clinical cure + markedly effective + effective) / Total number of cases × 100%. |
| Li Liang [31] | Overall response rate = (Number of cases with markedly effective + effective) / Total number of cases × 100%. |
| Wang Mi [33] | The efficacy evaluation was conducted with reference to the *Chinese Expert Consensus on Herpes Zoster*.  Clinical cure: Complete resolution of skin lesions with restoration of normal skin, disappearance of pruritus, or therapeutic index ≥ 95%.  Markedly effective: Partial resolution of skin lesions with significant relief of pruritus and no exudation; 70% ≤ Therapeutic index < 95%.  Effective: Local resolution of skin lesions with alleviated pruritus; 30% ≤ Therapeutic index < 70%.  Ineffective: No change in the resolution of skin lesions, with unchanged or aggravated symptoms, or therapeutic index < 30%.  Overall response rate = (Number of cases with clinical cure + markedly effective + effective) / Total number of cases × 100%. |
| Li Hui [34] | The efficacy evaluation criteria were determined with reference to the *TCM Diagnostic and Therapeutic Criteria for Diseases and Syndromes*.  Clinical cure: All herpes lesions crusted or shed, with complete disappearance of pain.  Markedly effective: Crusted area of herpes lesions ≥ 70%, with significant pain relief and no new vesicles.  Effective: Crusted area of herpes lesions ranging from 30% to 70%, with partial pain relief, though residual pain or a small number of new vesicles persisted.  Ineffective: Crusted area of herpes lesions < 30%, with insignificant or even aggravated pain, or the appearance of new vesicles.  Overall response rate = (Number of cases with clinical cure + markedly effective + effective) / Total number of cases × 100%. |
| Cao Lei [35] | The efficacy evaluation criteria were formulated based on the *TCM Diagnostic and Therapeutic Criteria for Diseases and Syndromes*.  Neuropathic pain evaluation:  Clinical cure: Complete disappearance of pain, with no abnormal sensations or radiating pain around the affected area.  Effective: Significant pain relief, though mild pain persists, accompanied by occasional pruritus or numbness in the affected area.  Ineffective: No significant pain relief or even aggravation of pain. The "clinical cure" and "effective" categories were combined to calculate the overall response rate.  Overall response rate = (Number of cases with clinical cure + effective) / Total number of cases × 100%. |
| Shi Jiajun [37] | Clinical cure: Complete or near-complete disappearance of primary symptoms and signs; therapeutic index ≥ 90%.  Markedly effective: Significant improvement in primary symptoms and signs; 70% ≤ Therapeutic index < 90%.  Effective: Obvious improvement in primary symptoms and signs; 30% ≤ Therapeutic index < 70%.  Ineffective: No significant improvement or aggravation of primary symptoms and signs; therapeutic index < 30%.  Therapeutic index = [(Pre-treatment TCM syndrome score − Post-treatment TCM syndrome score) / Pre-treatment TCM syndrome score] × 100%.  Overall response rate = (Number of cases with clinical cure + markedly effective + effective) / Total number of cases × 100%. |
| Meng Zhiyang [38] | The efficacy evaluation criteria were formulated with reference to the *TCM Diagnostic and Therapeutic Criteria for Diseases and Syndromes*.  Clinical cure: Complete resolution of herpes lesions, with disappearance of pain and accompanying symptoms.  Markedly effective: Resolution of herpes lesions ≥ 70%, with significant pain relief and a reduction in VAS score of > 2 points.  Effective: Resolution of herpes lesions ranging from 30% to 70%, with partial pain relief and a reduction in VAS score of 1–2 points.  Ineffective: Resolution of herpes lesions < 30%, with persistent severe pain and a reduction in VAS score of < 1 point.  Overall response rate = (Number of cases with clinical cure + markedly effective + effective) / Total number of cases × 100%. |
| Wang Gang [40] | Clinical cure: Complete crusting of vesicles with complete or near-complete shedding of crusts, and complete disappearance of clinical symptoms such as pain.  Markedly effective: At least 80% of vesicles crusted, with significant relief or near-complete disappearance of pain at the lesion site.  Effective: Crusted area of vesicles ranging from 50% to 79%, with partial pain relief at the lesion site.  Ineffective: Crusted area of vesicles < 50%, with no pain relief or aggravated pain.  Overall response rate = (Number of cases with clinical cure + markedly effective + effective) / Total number of cases × 100%. |
| Cui Jing [41] | The efficacy evaluation criteria were formulated with reference to the *TCM Diagnostic and Therapeutic Criteria for Diseases and Syndromes*.  Clinical cure: Complete disappearance of symptoms, including vesicles and pain.  Markedly effective: Resolution area of vesicles > 80%, with near-complete disappearance of pain.  Effective: Resolution area of vesicles ranging from 60% to 80%, with significant pain relief.  Ineffective: Resolution area of vesicles < 60%, with no significant pain relief.  Overall response rate = (Number of cases with clinical cure + markedly effective + effective) / Total number of cases × 100%. |
| Cai Xiaoce [43] | The efficacy evaluation criteria were determined based on the TCM Syndrome Diagnostic and Therapeutic Criteria promulgated by the National Administration of Traditional Chinese Medicine.  Clinical cure: Complete resolution of skin lesions and disappearance of clinical symptoms, with no residual pain one month later.  Improved: Resolution area of skin rash of approximately 30%, with significant pain relief.  Ineffective: Resolution area of skin rash < 30%, with persistent pain.  Overall response rate = (Number of cases with clinical cure + improved) / Total number of cases × 100%. |
| Pang Jian'en [44] | Markedly effective: Complete resolution of skin rash, with a VAS pain score of < 3.  Effective: Resolution area of skin rash > 50%, with a VAS pain score ranging from 3 to 5.  Ineffective: Neither the resolution area of the skin rash nor the VAS pain score met the criteria for "effective".  Overall response rate = (Number of cases with markedly effective + effective) / Total number of cases × 100%. |
| Li Zhiquan [45] | The efficacy evaluation criteria were formulated with reference to the *TCM Diagnostic and Therapeutic Criteria for Diseases and Syndromes*.  Clinical cure: Successful completion of the treatment course, with crusting of herpes lesions and no development of postherpetic neuralgia (PHN).  Markedly effective: Resolution of herpes lesions > 60%, with most lesions crusted and pain nearly completely disappeared.  Effective: Resolution of herpes lesions > 30%, with partial crusting and mild pain.  Ineffective: Failure to complete the treatment, resolution of skin rash < 30% or no resolution, formation of new vesicles, or development of PHN.  Overall response rate = (Number of cases with clinical cure + markedly effective + effective) / Total number of cases × 100%. |
| Xing Yukui [46] | Markedly effective: Complete disappearance of skin rash, clinical symptoms, and pain.  Effective: Resolution of skin rash ≥ 30%, with significant improvement in pain and clinical symptoms.  Ineffective: Resolution of skin rash ≤ 29%, with no significant change or aggravation of pain and clinical symptoms.  Overall response rate = (Number of cases with markedly effective + effective) / Total number of cases × 100%. |
| Bai Meng [47] | Overall response rate = (Number of cases with markedly effective + effective) / Total number of cases × 100%. |
| Wei Fan [48] | The efficacy evaluation criteria were formulated with reference to the *TCM Diagnostic and Therapeutic Criteria for Diseases and Syndromes* promulgated by the National Administration of Traditional Chinese Medicine.  Clinical cure: Complete resolution of skin rash and disappearance of clinical signs, with no residual pain.  Improved: Resolution of skin rash of approximately 30%, with significant pain relief.  Unhealed: Resolution of skin rash < 30%, with persistent pain.  Response rate = (Number of cases with clinical cure + improved) / Total number of cases × 100%. |
| Tong Laisheng [49] | The efficacy evaluation criteria were formulated with reference to the *Mongolian Medicine Diagnostic and Therapeutic Criteria for Diseases and Syndromes*.  Clinical cure: Complete resolution of skin rash and disappearance of clinical signs, with no residual pain.  Effective: Resolution of skin rash > 30%, with significant pain relief.  Ineffective: Resolution of skin rash < 30%, with no significant pain relief.  Overall response rate = (Number of cases with clinical cure + effective) / Total number of cases × 100%. |
| Li Hu [50] | Clinical cure: Complete crusting of all herpes lesions, with complete disappearance of pain.  Markedly effective: Crusted area of herpes lesions ≥ 70%, with significant pain relief and no new vesicles.  Improved: Crusted area of herpes lesions ranging from 30% to 70%, with mild pain or a small number of new vesicles.  Ineffective: Crusted area of herpes lesions ≤ 30%, or the appearance of new vesicles with no pain relief.  Overall response rate = (Number of cases with clinical cure + markedly effective) / Total number of cases × 100%. |
| Chen Wei [51] | The efficacy evaluation criteria were formulated with reference to the *Shanghai Routine for TCM Diagnosis and Treatment of Diseases and Syndromes*.  Clinical cure: Complete resolution of skin rash and disappearance of clinical signs, with no residual pain.  Improved: Resolution of skin rash > 30%, with significant pain relief.  Unhealed: Resolution of skin rash < 30%, with persistent pain.  Overall response rate = (Number of cases with clinical cure + improved) / Total number of cases × 100%. |
| Gao Jianzhong [52] | The efficacy evaluation criteria were formulated with reference to the *TCM Diagnostic and Therapeutic Criteria for Diseases and Syndromes*.  Clinical cure: Complete resolution of skin rash and disappearance of clinical signs, with no residual pain.  Improved: Resolution of skin rash > 30%, with significant pain relief.  Unhealed: Resolution of skin rash < 30%, with persistent pain.  Overall response rate = (Number of cases with clinical cure + improved) / Total number of cases × 100%. |
| Zhao Bohua [53] | The efficacy evaluation criteria were formulated with reference to the *TCM Diagnostic and Therapeutic Criteria for Diseases and Syndromes*.  The therapeutic efficacy was evaluated based on the therapeutic index (n), calculated as: n = [(Total pre-treatment score − Total post-treatment score) / Total pre-treatment score] × 100%.  Clinical cure: Complete crusting of skin rash and disappearance of pain; n ≥ 95%.  Markedly effective: Most of the skin rash crusted, with significant pain relief; 70% ≤ n < 95%.  Effective: Partial crusting of skin rash, with significant pain relief; 30% ≤ n < 70%.  Ineffective: Basically no change in symptoms; n < 30%.  Overall response rate = (Number of cases with clinical cure + markedly effective + effective) / Total number of cases × 100%. |
| Pan Dong [54] | Clinical cure: Complete crusting of skin rash and disappearance of pain; n ≥ 95%.  Markedly effective: Most of the skin rash crusted, with significant pain relief; n ≥ 70%.  Effective: Partial crusting of skin rash, with pain relief; 30% ≤ n < 70%.  Ineffective: Basically no change in symptoms; n < 30%.  Therapeutic index (n) = [(Total pre-treatment score − Total post-treatment score) / Total pre-treatment score] × 100%.  Overall response rate = (Number of cases with clinical cure + markedly effective + effective) / Total number of cases × 100%. |
| Liu Haiying [55] | Clinical cure: Stabbing pain disappeared within 7–10 days, with crusting, desiccation, and shedding of skin lesions, and complete resolution of all symptoms.  Markedly effective: Stabbing pain disappeared within 11–15 days, with shedding of crusts and restoration of normal skin.  Ineffective: Vesicles became shriveled within 16–20 days.  Overall response rate = (Number of cases with clinical cure + markedly effective) / Total number of cases × 100%. |
| Shao Suju [56] | The efficacy evaluation criteria were formulated based on the *TCM Diagnostic and Therapeutic Criteria for Diseases and Syndromes* promulgated by the National Administration of Traditional Chinese Medicine.  Clinical cure: Complete resolution of skin rash and disappearance of clinical signs, with no residual pain.  Improved: Resolution of skin rash of approximately 30%, with significant pain relief.  Unhealed: Resolution of skin rash < 30%, with persistent pain.  Overall response rate = (Number of cases with clinical cure + improved) / Total number of cases × 100%. |
| Song Suyan [57] | The efficacy evaluation criteria were formulated based on the *Clinical Diagnostic Criteria and Standards for Cure and Improvement of Diseases*.  Clinical cure: Complete or near-complete resolution of herpes lesions at the affected site, with disappearance of pain.  Markedly effective: Most of the herpes lesions crusted, with near-complete disappearance of pain at the affected site.  Effective: Resolution of herpes lesions ranging from 20% to 50%, with alleviated pain at the affected site.  Ineffective: Resolution of herpes lesions < 20%, with no pain relief.  Postherpetic neuralgia (PHN): Persistent pain after the resolution of herpes lesions at the affected site.  Overall response rate = (Number of cases with clinical cure + markedly effective + effective) / Total number of cases × 100%. |
| Wu Huajun [58] | The efficacy evaluation criteria were formulated with reference to the *TCM Diagnostic and Therapeutic Criteria for Diseases and Syndromes* promulgated by the National Administration of Traditional Chinese Medicine in 1994.  Clinical cure: Complete resolution of skin rash and disappearance of clinical signs and pain.  Improved: Resolution of skin rash > 30%, with significant pain relief.  Ineffective: Resolution of skin rash < 10%, with no pain relief.  Overall response rate = (Number of cases with clinical cure + improved) / Total number of cases × 100%. |
| Zhang Yalan [59] | Clinical cure: Complete disappearance of herpes lesions and pain.  Markedly effective: Partial resolution of herpes lesions, with alleviated pain and other symptoms.  Ineffective: No improvement or aggravation of symptoms.  Overall response rate = (Number of cases with clinical cure + markedly effective) / Total number of cases × 100%. |
| Xu Lan [60] | Clinical cure: Therapeutic index = 100%.  Markedly effective: Therapeutic index decreased by 60%–99%.  Improved: Therapeutic index decreased by 30%–59%.  Ineffective: Therapeutic index decreased by < 30%.  Therapeutic index = [(Pre-treatment score − Post-treatment score) / Pre-treatment score] × 100%.  Overall response rate = (Number of cases with clinical cure + markedly effective) / Total number of cases × 100%. |

Supplementary Figure 1. Regression Analysis Results of VAS by Treatment Course

Supplementary Figure 2. Meta-Regression Results of VAS Scores by Publication Year

Supplementary Figure 3. Meta-Regression Results of VAS Scores by Sample Size

Supplementary Figure 4. Sensitivity Analysis of VAS Scores After Excluding Studies with High Risk of Bias

Supplementary Figure 5. Egger’s Test Result Plot for VAS Scores

Supplementary Figure 6. Egger Plot for VAS

Supplementary Figure 7. Trim-and-Fill Results of VAS Funnel Plot

Supplementary Figure 8. Trim-and-Fill of VAS Funnel Plot

Supplementary Figure 9. Sensitivity Analysis of the Overall Response Rate

Supplementary Figure 10. Egger’s Test Result Plot for the Overall Response Rate

Supplementary Figure 11. Egger Plot for Overall Response Rate

Supplementary Figure 12. Meta-regression Analysis of Time to Cessation of Vesicles by Treatment Course

Supplementary Figure 13. Meta-regression Analysis of Time to Cessation of Vesicles by Publication Year

Supplementary Figure 14. Meta-regression Analysis of Time to Cessation of Vesicles by Sample Size

Supplementary Figure 15. Sensitivity Analysis of Time to Cessation of Vesicles After Excluding Studies with High Risk of Bias

Supplementary Figure 16. Funnel Plot for Time to Cessation of Vesicles

Supplementary Figure 17. Trim-and-Fill Results of the Funnel Plot for Time to Cessation of Vesicles

Supplementary Figure 18. Trim-and-Fill Analysis of the Funnel Plot for Time to Cessation of Vesicles

Supplementary Figure 19. Meta-regression Analysis of Time to Crust Formation by Treatment Course

Supplementary Figure 20. Meta-regression Analysis of Time to Crust Formation by Publication Year

Supplementary Figure 21. Meta-regression Analysis of Time to Crust Formation by Sample Size

Supplementary Figure 22. Sensitivity Analysis of Time to Crust Formation After Excluding Studies with High Risk of Bias

Supplementary Figure 23. Funnel Plot for Time to Crust Formation

Supplementary Figure 24. Trim-and-Fill Results of the Funnel Plot for Time to Crust Formation

Supplementary Figure 25. Trim-and-Fill Analysis of the Funnel Plot for Time to Crust Formation

Supplementary Figure 26. Sensitivity Analysis of the Incidence of Postherpetic Neuralgia After Excluding Studies with High Risk of Bias

Supplementary Figure 27. Funnel Plot for the Incidence of Postherpetic Neuralgia

Supplementary Figure 28. Trim-and-Fill Analysis of the Funnel Plot for the Incidence of Postherpetic Neuralgia (Adjusted)

Supplementary Figure 29. Trim-and-Fill Analysis of the Funnel Plot for the Incidence of Postherpetic Neuralgia (Unadjusted)
